# Qingfei Jiedu decoction inhibits PD-L1 expression in lung adenocarcinoma based on network pharmacology analysis, molecular docking and experimental verification

**DOI:** 10.3389/fphar.2022.897966

**Published:** 2022-08-22

**Authors:** Junjie Pan, Hongkuan Yang, Lihong Zhu, Yafang Lou, Bo Jin

**Affiliations:** ^1^ Department of Pulmonary and Critical Care Medicine, Hangzhou Hospital of Traditional Chinese Medicine (Dingqiao District), Hangzhou, Zhejiang, China; ^2^ Department of Pulmonary and Critical Care Medicine, Hangzhou Hospital of Traditional Chinese Medicine, Hangzhou, Zhejiang, China; ^3^ Respiratory Intensive Care Unit, The People’s Hospital of Gaozhou, Maoming, Guangdong, China; ^4^ College of Life Science, Zhejiang Chinese Medical University, Hangzhou, Zhejiang, China

**Keywords:** Qingfei Jiedu decoction, lung adenocarcinoma, programmed cell death ligand-1, CD8^+^PD-1^+^T, network pharmacology, molecular docking

## Abstract

**Objective:** We aim at investigating the molecular mechanisms through which the Qingfei Jiedu decoction (QFJDD) regulates PD-L1 expression in lung adenocarcinoma (LUAD).

**Methods:** Bioactive compounds and targets of QFJDD were screened from TCMSP, BATMAN-TCM, and literature. Then, GeneCard, OMIM, PharmGKB, Therapeutic Target, and DrugBank databases were used to identify LUAD-related genes. The protein-protein interaction (PPI) network was constructed using overlapping targets of bioactive compounds in LUAD with the Cytoscape software and STRING database. The potential functions and pathways in which the hub genes were enriched by GO, KEGG, and DAVID pathway analyses. Molecular docking of bioactive compounds and key genes was executed *via* AutoDock Vina. Qualitative and quantitative analyses of QFJDD were performed using UPLC-Q-TOF-MS and UPLC. Expressions of key genes were determined by qRT-PCR, immunoreactivity score (IRS) of PD-L1 was assessed by immunohistochemistry (IHC), while the CD8^+^PD-1^+^T% derived from spleen tissues of Lewis lung cancer (LLC) bearing-mice was calculated using flow cytometry (FCM).

**Results:** A total of 53 bioactive compounds and 288 targets of QFJDD as well as 8151 LUAD associated genes were obtained. Further, six bioactive compounds, including quercetin, luteolin, kaempferol, wogonin, baicalein, and acacetin, and 22 hub genes were identified. The GO analysis showed that the hub genes were mainly enriched in DNA or RNA transcription. KEGG and DAVID pathway analyses revealed that 20 hub genes were primarily enriched in virus, cancer, immune, endocrine, and cardiovascular pathways. The EGFR, JUN, RELA, HIF1A, NFKBIA, AKT1, MAPK1, and MAPK14 hub genes were identified as key genes in PD-L1 expression and PD-1 checkpoint pathway. Moreover, ideal affinity and regions were identified between core compounds and key genes. Notably, QFJDD downregulated EGFR, JUN, RELA, HIF1A, NFKBIA, and CD274 expressions (*p* < 0.05), while it upregulated AKT1 and MAPK1 (*p* < 0.05) levels in A549 cells. The PD-L1 IRS of LLC tissue in the QFJDD high dose (H_d_) group was lower than model group (*p* < 0.01). CD8^+^PD-1^+^T% was higher in the QFJDD H_d_ group than in normal and model groups (*p* < 0.05).

**Conclusion:** QFJDD downregulates PD-L1 expression and increases CD8^+^PD-1^+^T% *via* regulating HIF-1, EGFR, JUN and NFκB signaling pathways. Therefore, QFJDD is a potential treatment option for LUAD.

## 1 Introduction

According to the global cancer data 2020, lung cancer, especially small cell lung cancer and non-small cell lung cancer (NSCLC), is the most lethal cancer (Sung et al., 2021). In China, among all the histological subtypes of NSCLC, lung adenocarcinoma (LUAD) is the most prevalent. Lung cancer is often diagnosed in the late stages and therefore has a poor overall survival rate (Duma et al., 2019). Anti-tumor drugs have been shown to improve the prognosis of lung cancer (Wu et al., 2021). Moreover, the advent of programmed cell death-1 (PD-1)/programmed cell death ligand-1 (PD-L1) has resulted in better prognostic outcomes of NSCLC patients (Jain et al., 2018). However, due to the high heterogeneity of NSCLC and the varying degrees of T cell infiltrations into the tumor microenvironment, the prognostic outcomes for patients remains poor. Therefore, there is a need to identify new therapeutic agents for NSCLC.

For thousands of years, Traditional Chinese medicine (TCM) has been used as a complementary and alternative medicine (Cyranoski, 2018) to treat various diseases, including malignancies (Zhang et al., 2021). In recent years, TCM are combined with chemotherapy, targeted therapies, or immune checkpoint inhibitors to treat various cancers (Su et al., 2020). However, the mechanisms of action of TCM prescriptions have not been fully established. Over the years, network pharmacology have been used to study the pharmacological mechanisms of famous prescriptions recorded in ancient TCM books, but, the mechanisms of empirical prescriptions have rarely been explored. Qingfei Jiedu Decoction (QFJDD) is an empirical prescription that has been used for the complementary intervention of lung cancer by our medical team (Pan et al., 2020a). This prescription is prepared from Scutellariae Barbatae Herba (Banzhilian, BZL), Lobeliae Chinensis Herba (Banbianlian, BBL), Hedyotis Diffusae Herba (Baihuasheshecao, BHSSC), Herba Solani Lyrati (Baimaoteng, BMT), Solanum Nigrum (Longkui, LK), and Coicis Semen (Yiyiren, YYR) (Table 1). Previously, we showed that QFJDD inhibits Lewis lung cancer (LLC) cell proliferations and down-regulates PD-L1 expression in tumor tissues (Pan et al., 2020b). However, the active ingredients and pharmacological mechanisms of QFJDD have not been established.

**TABLE 1:** The composition of QFJDD.

Chinese name	Pharmaceutical name	Botanical plant name
Ban Zhi Lian	Scutellariae Barbatae Herba	*Scutellaria barbate* D. Don
Ban Bian Lian	Lobeliae Chinensis Herba	*Lobelia chinensis* Lour
Bai Hua She She Cao	Hedyotis Diffusae Herba	*Oldenlandia diffusa* (Wild.) Roxb
Bai Mao Teng	Herba Solani Lyrati	*Aristolochia mollissima* Hance
Long Kui	Solanum Nigrum	*Solanum nigrum* L
Yi Yi Ren	Coicis Semen	*Coix lacryma-jobi* L. var. Ma-yuen. (Roman.) Stapf

**FIGURE 1:**
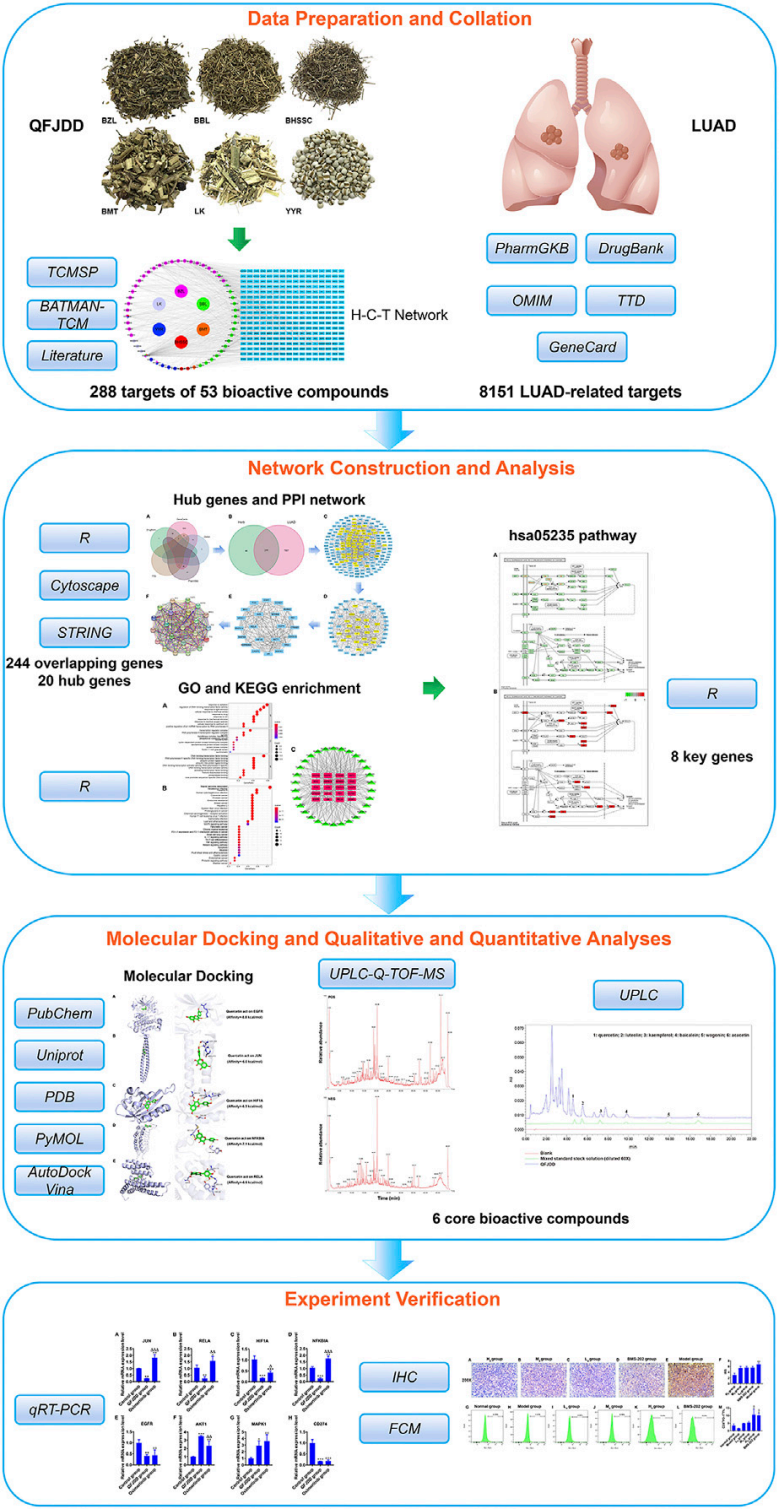
The flowchart showing the molecular mechanisms by which QFJDD inhibits PD-L1 expression in LUAD.

In this study, network pharmacology and experimental verification were performed to investigate the mechanisms through which QFJDD inhibits PD-L1 expression in LUAD (Figure 1). First, bioactive compounds and targets of QFJDD against LUAD were searched and screened. Then, hub genes, biological functions, and the key signaling pathways associated with QFJDD were identified by network construction and analysis. Finally, the results were validated through cellular and animal experiments.

## 2 Materials and methods

### 2.1 Data preparation and collation

#### 2.1.1 Bioactive compounds and action targets of Qingfei Jiedu decoction

At first, the Traditional Chinese Medicine Systems Pharmacology database and Analysis Platform (TCMSP) (Ru et al., 2014), Bioinformatics Analysis Tool for Molecular mechANism of Traditional Chinese Medicine (BATMAN-TCM) (Liu et al., 2016a), and the published literature were used to screen bioactive compounds in BHSSC, BZL, BBL, BMT, YYR, and LK. Bioactive compounds from BATMAN-TCM database were numbered successively by BATMAN001, BATMAN002, etc. Then, the SwissADME web tool (Daina et al., 2017) was used to detect the oral bioavailability (OB), drug-likeness (DL) and pharmacokinetics of each bioactive compounds. The screening criteria were OB ≥ 30% and DL ≥ 0.18 (Tu et al., 2021). The screening criterion of core bioactive compounds was degree >24 (Wang et al., 2021). Moreover, the potential targets of bioactive compounds in QFJDD were searched from TCMSP, BATMAN-TCM, and SwissTargetPrediction (Daina et al., 2019). Finally, the duplicate bioactive compounds and targets were removed to obtain the herb-gene text file.

#### 2.1.2 Lung adenocarcinoma related genes

LUAD related genes were searched using the keyword “lung adenocarcinoma” in various databases, including GeneCard (https://www.genecards.org/) (Stelzer et al., 2016), Online Mendelian Inheritance in Man (OMIM, https://omim.org/) (Amberger and Hamosh, 2017), PharmGKB (https://www.pharmgkb.org/) (Gong et al., 2021), Therapeutic Target (TTD, http://db.idrblab.net/ttd/) (Wang et al., 2020) and DrugBank (https://www.drugbank.ca/) (Wishart et al., 2018). Target genes were limited to *Homo sapiens*. Furthermore, Venn diagrams were plotted using R packages to display overlapping genes between LUAD and QFJDD.

### 2.2 Network construction and analysis

#### 2.2.1 Herb-compound-target network

The herb-gene text file was processed using Strawberry Perl programming language to obtain files of the all-net-list, col-herb-list, col-gene-list, col-id-list, id-pie-input-list and item-types-input-list. Afterward, the Cytoscape 3.9.1 software (Shannon et al., 2003) was used to establish the herb-compound-target (H-C-T) network.

#### 2.2.2 Construction of the protein-protein interaction and hub genes network

A total of 244 herb-LUAD genes were submitted to the Cytoscape 3.9.1 software to identify hub genes. Then, the STRING 11.5 database (https://www.string-db.org/) (Szklarczyk et al., 2021) was used to construct the PPI network with a confidence score >0.9. Targets were limited to the *Homo sapiens* species.

#### 2.2.3 Functional and pathway enrichment analyses

Gene ontology (GO) (Gaudet et al., 2021), Kyoto Encyclopedia of Genes and Genomes (KEGG) (Kanehisa et al., 2017) and Database for Annotation, Visualization, and Integrated Discovery (DAVID) (Dennis et al., 2003) pathway analyses were performed to determine the functions and pathways in which the hub genes were enriched. These analyses were carried out in R 4.1.1 software.

### 2.3 Molecular docking analysis

Initially, 3D molecular structures of six small molecule ligands were downloaded from the PubChem (https://pubchem.ncbi.nlm.nih.gov/) website (Kim et al., 2021). Subsequently, Uniprot IDs of seven protein receptor conformations corresponding to key genes were retrieved from the Protein Data Bank (PDB, https://www.rcsb.org/) database (Velankar et al., 2021). Screening parameters were: 1) X-crystal diffraction and crystal resolution<3Å; 2) Protein structures of *Homo sapiens*. Based on PyMOL version 2.5 software, the following options were carried out: assignation of bond orders, addition of hydrogens, creation of zero-order bonds to metals, creation of disulfide bonds, deletion of waters beyond 5Å from het groups and calculation of the molecular activity pockets. Molecular docking simulation was executed using AutoDock Vina version 1.1.2 version software (Seeliger and de Groot, 2010). Binding energy was determined from affinity.

### 2.4 Experimental verification of bioinformatic results

#### 2.4.1 Preparation of Qingfei Jiedu decoction

The QFJDD was prepared from BHSSC (Origin Zhejiang, Hangzhou Huadong TCM pieces Co., Ltd.), BZL (Origin Zhejiang, Hangzhou Huadong TCM pieces Co., Ltd.), BBL (Origin Zhejiang, Zhejiang Yingte TCM pieces Co., Ltd.), BMT (Origin Zhejiang, Zhejiang Yingte TCM pieces Co., Ltd.), YYR (Origin Guizhou, Zhejiang Zuoli Baicao TCM pieces Co., Ltd.) and LK (Origin Zhejiang, Zhejiang Zuoli Baicao TCM pieces Co., Ltd.) in weight ratios of 5:5:5:5:10:2. Herbs were purchased from the Hangzhou Hospital of Traditional Chinese Medicine pharmacy. First, 672 g of the herbs were soaked in 3,000 ml distilled water for 30 min, boiled at 100°C for 30 min, centrifuged at 1847 (×g) for 5 min and filtered twice via a 0.22 µm filter. A total of 777.8 ml QFJDD (0.864 g/ml) was obtained and stored at 4°C.

#### 2.4.2 Qualitative and quantitative analyses of six bioactive compounds in Qingfei Jiedu decoction

Ultra-Performance Liquid Chromatography Quadrupole Time-of-Flight Mass Spectrometry (UPLC-Q-TOF-MS) was used to isolate and identify six bioactive compounds of QFJDD which were extracted and characterized using UPLC-Q-TOF-MS on the Waters^®^ SYNAPT^®^ G2-Si system with a waters CORTECS UPLC T3 Column (2.1 mm × 100 mm, 1.67 μm). Solvent A (acetonitrile) and solvent B (0.1% formic acid-H_2_O) were used for gradient elution as shown in Supplementary Table S1. The MS, which was run in both positive and negative electrospray ionization, parameters are shown in Supplementary Table S2. Quercetin, luteolin, kaempferol, wogonin, baicalein, and acacetin were purchased from Chengdu herbpurify Co., Ltd, and the purity of all standards has met the analytical requirements. The MS instrumentation and data acquisition were conducted using the Masslynx^®^ V4.1 software.

Moreover, a volume of 2.8 ml QFJDD was accurately taken into a volumetric flask and fixed the volume to 25 ml with ultra-pure water. After that, the supernatant was obtained by centrifugation at 1847 (×g) for 5 min and filtered *via* a 0.45 µm filter membrane. Quercetin, luteolin, kaempferol, wogonin, baicalein and acacetin solutions were prepared in methanol at final concentrations of 0.1088 mg/ml, 0.0856 mg/ml, 0.1072 mg/ml, 0.1440 mg/ml, 0.1108 mg/ml, and 0.1164 mg/ml, separately. A further 100 μl of each standard solution was taken into a volumetric flask and diluted with 900 μl methanol. UPLC was used to quantify six bioactive compounds of QFJDD which were analyzed using UPLC on the Waters ACQUITY UPLC^®^ H-Class system with a ACQUITY UPLC BEH C18 Column (2.1 mm × 100 mm, 1.7 μm) (Supplementary Figure S1 **)**. Solvent A (0.2% phosphoric acid-distilled water) and solvent B (methanol) were used for gradient elution as shown in Supplementary Table S3. Standard curves were constructed as shown in Supplementary Table S4 and the amount of six bioactive compounds in QFJDD was calculated. The setting parameters were as follows: column temperature = 40°C; injection volume = 5 μl; wavelength = 350 nm.

#### 2.4.3 Cell and animal cultures

The A549 and LLC cell lines were purchased from the Stem Cell Bank, Chinese Academy of Sciences (Shanghai, China). The A549 cells were cultured in F-12K (Invitrogen) with 10% fetal bovine serum (FBS, Gibco) and 1% Glutamax (Invitrogen). Incubation was done at 37°C in a 5% CO_2_ atmosphere. The LLC cells were cultured in DMEM (Gibco) with 10% FBS and incubated at 37°C in a 5% CO_2_ atmosphere. Six-week-old male Sprague-Dawley (SD) rats (*n* = 20, 200 ± 10 g) and five-week-old male C57BL/6 mice (*n* = 25, 14–16 g) of SPF grade were purchased from the Shanghai SLAC Laboratory Animal Co., Ltd. [license No. SCXK (Shanghai) 2017-0005]. Animals were maintained at the Zhejiang Chinese Medical University Laboratory Animal Center (22 ± 2°C and 40%–70% relative humidity with a 12-h light/12-h dark cycle) with *ad libitum* access to food and water.

#### 2.4.4 Preparation of Qingfei Jiedu decoction-containing serum

After 5 days of adaptive feeding, SD rats were randomized into two groups (*n* = 10). Equivalent doses between humans and rats were converted according to body surface area (Nair and Jacob, 2016). Thus, the control group was administered with 0.9% saline (1 ml/100 g) while the QFJDD group received 8.64 g/kg of QFJDD. Rats were intragastrically administered with drugs once daily for seven consecutive days (Li et al., 2020). An hour after the final drug administration, rats were anesthetized by 3% sodium pentobarbital via peritoneal injection after which whole blood was collected by cardiac puncture. Blood samples were allowed to stand at room temperature for 2 h, and afterwards the serum was prepared by centrifugation at 1847 (×g) for 10 min at 4°C. After that, the complement was inactivated at 56°C for 60 min. Serum was passed through a 0.22 μm filter to remove bacteria and stored at −80°C.

#### 2.4.5 Establishment of Lewis lung cancer-bearing mouse model and drug intervention

After 7 days of adaptive feeding, C57BL/6 mice were randomized into six groups, each containing five mice. After that, 0.2 ml of 1×10^7^/ml LLC cell suspension was subcutaneously inoculated into the right axilla of C57BL/6 mice, apart from the normal group. When the tumor diameter reached 0.5 cm, drugs were initiated and administered for 14 days. The high dose (H_d_, 57.6 g/kg/d), medium dose (M_d_, 28.8 g/kg/d) and low dose (L_d_, 14.4 g/kg/d) groups were administered with once-daily doses of QFJDD via oral gavage. The PD-L1 inhibitor group (BMS-202, MedChemExpress, United States, 17.2 mg/kg) received once-weekly injections *via* the caudal vein. In addition, model and normal groups received a once-daily oral gavage of 0.9% sterile saline. At 24 h after the last dose, tumor and the spleen tissues were aseptically harvested. The equivalent dose between human and mouse was determined based on body surface area (Nair and Jacob, 2016). The doses of QFJDD in different groups were based on our previous study (Pan et al., 2020b).

#### 2.4.6 Quantitative real-time PCR

The A549 cells were divided into control (10% rat serum), Osimertinib (6 μM, MedChemExpress, United States), and QFJDD (10% QFJDD-containing serum) groups. After 48 h of incubation with serum or Osimertinib, total RNA was extracted using the TRIzol. The PrimeScript™ TM RT Master Mix (Takara, Dalian, China) was used for cDNA synthesis for reverse transcription PCR under the following conditions: 37°C for 15 min, 85°C for 5 s and stored at 4°C. The SYBR^®^ Prime Ex Taq TM II (Tli RNaseH plus) (Takara, Dalian, China) was used for Real-time fluorescence quantitative PCR under the following parameters: 40 cycles at 95°C for 2 min, 55°C for 30 s, 72°C for 30 s. Primer sequences used are shown Supplementary Table S5.

#### 2.4.7 Immunohistochemistry

Tumor tissues were fixed in 4% paraformaldehyde for 24 h and embedded in paraffin. The tissues were then sectioned into 4 µm pieces. After antigen retrieval, sections were incubated with monoclonal rabbit antibodies against PD-L1 (eBioscience, United States) to determine PD-L1 expression. Subsequently, sections were treated with horseradish peroxidase-conjugated secondary antibodies. Visualization was done using diaminobenzidine while counterstaining was done with hematoxylin. Immunoreactivity was evaluated using the immunoreactivity score (IRS) (Lee et al., 2020). IRS = staining intensity (SI)×positive percentage (PP). The SI was divided into 0 (negative), 1 (weak), 2 (moderate) and 3 (strong). The PP had five levels, including 0 (negative, ≤10%), 1 (11%–25%), 2 (26%–50%), 3 (51%–75%) and 4 (76%–100%).

#### 2.4.8 Flow cytometry

Connective tissues of the spleen were aseptically removed from mice and washed in precooled PBS. The spleen was punctured repeatedly with a sterile needle after which PBS was injected to drain the lymphocytes. After that, tissues were filtered using a 200-mesh screen, washed twice using PBS, and centrifuged at 1,200 rpm for 5 min to obtain a single cell suspension of 1×10^6^/ml. Subsequently, 100 μl of the single cell suspension was added into a flow tube. 2 μl of CD3 (eBioscience, United States), 2 μl of CD8 (eBioscience, United States), and 2 μl PD-1 (eBioscience, United States) labeled antibody were added to the experimental tube, while the corresponding same type of control antibody was added to the control tube, and incubated at room temperature in dark for 15 min. 500 μl red blood cell lysis buffer (Sigma Aldrich, United States) was then added and incubated at room temperature for 10 min. 5 ml precooled PBS was added to cells and centrifuged at 1,200 rpm for 5 min, after which the supernatant was discarded. After centrifugation and re-suspension, the percentage of CD8^+^PD-1^+^T was determined by flow cytometry (Agilent Novocyte, United States).

#### 2.4.9 Statistical analysis

Data are presented as mean ± SD. Statistical analyses were performed using SPSS 25.0 software. Expressions of key genes in A549 cells were compared by one-way analysis of variance (ANOVA) (Bewick et al., 2004). Differences in PD-L1 IRS in subcutaneous tumor tissues between multiple groups were determined by Kruskal-Wallis nonparametric analysis of variance with multiple group comparisons (Theodorsson-Norheim, 1986). Comparison of CD8^+^PD-1^+^T% among multiple groups were performed by Kruskal-Wallis nonparametric analysis of variance. *p*-value<0.05 was considered statistically significant.

## 3 Results

### 3.1 Bioactive compounds and candidate targets network of Qingfei Jiedu decoction

**FIGURE 2:**
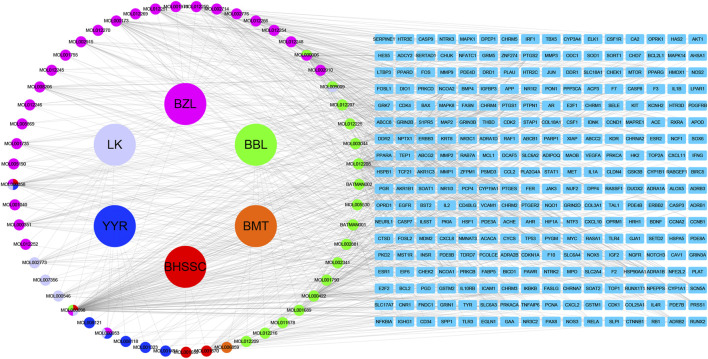
Herb-Compound-Target Network.

Based on the filter criteria of OB ≥ 30% and DL ≥ 0.18, preliminary screening generated 4 compounds in BHSSC, 27 compounds in BZL, 18 compounds in BBL, 1 compound in BMT, 6 compounds in YYR and 7 compounds in LK. After removing duplicate values, 53 bioactive compounds and 288 targets were obtained (Figure 2, Supplementary Table S6). Moreover, six core bioactive compounds, including quercetin, luteolin, kaempferol, wogonin, baicalein, and acacetin were identified with the screening criterion of degree>24 (Table 2). Pharmacokinetic parameters are presented in Table 3.

**TABLE 2:** Six core bioactive compounds of QFJDD.

MOL ID	CID	Compound	Molecular formula	Degree	2D structure
MOL000098	5280343	quercetin	C_15_H_10_O_7_	130	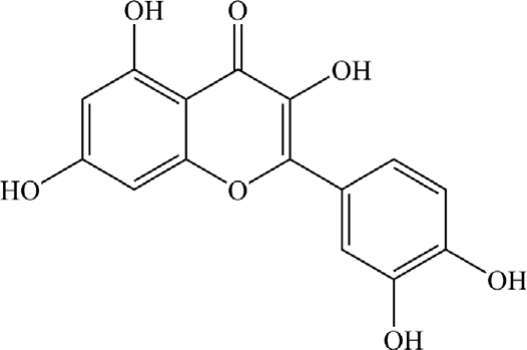
MOL000006	5280445	luteolin	C_15_H_10_O_6_	54	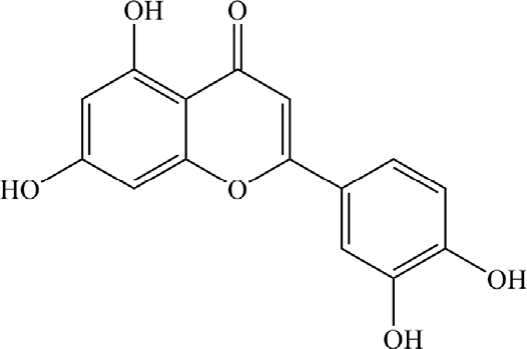
MOL000422	5280863	kaempferol	C_15_H_10_O_6_	50	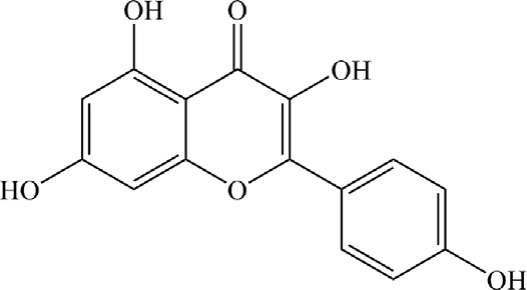
MOL000173	5281703	wogonin	C_16_H_12_O_5_	39	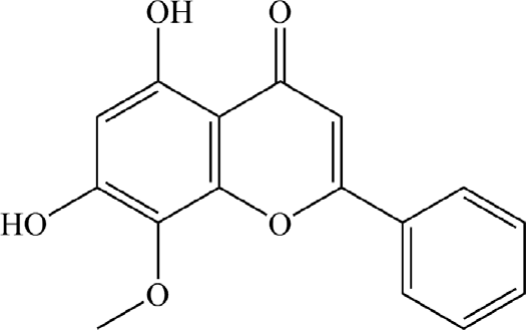
MOL002714	5281605	baicalein	C_15_H_10_O_5_	33	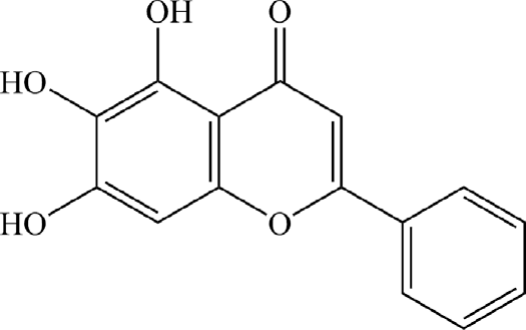
MOL001689	5280442	acacetin	C_16_H_12_O_5_	25	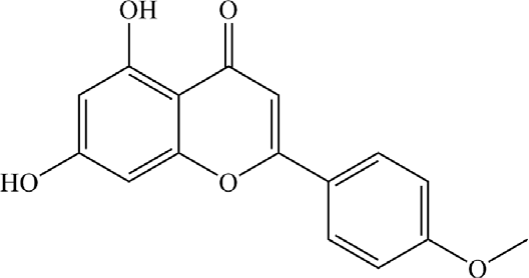

MOL ID, molecular ID from TCMSP database; CID, Compound CID from PubChem database. The 2D structures of the six core bioactive compounds were drawn by ChemBioDraw Ultra 14.0 software.

**TABLE 3:** Pharmacokinetic parameters of six core bioactive compounds.

Pharmacokinetics	Quercetin	Luteolin	Kaempferol	Wogonin	Baicalein	Acacetin
GI absorption	High	High	High	High	High	High
BBB permeant	No	No	No	Yes	No	Yes
P-gp substrate	No	No	No	No	Yes	No
CYP1A2 inhibitor	Yes	Yes	Yes	Yes	Yes	Yes
CYP2C19 inhibitor	No	No	No	Yes	No	Yes
CYP2C9 inhibitor	No	No	No	No	No	No
CYP2D6 inhibitor	Yes	Yes	Yes	No	No	No
CYP3A4 inhibitor	Yes	Yes	Yes	Yes	Yes	Yes
Log Kp (skin permeation, cm/s)	−7.05	−6.25	−6.70	−6.12	−6.17	−6.02

GI, absorption, Gatrointestinal absorption; BBB, permeant, according to the yolk of the BOILED-Egg. All pharmacokinetic parameters were detected using SwissADME web tool.

### 3.2 The protein-protein interaction network and hub genes

**FIGURE 3:**
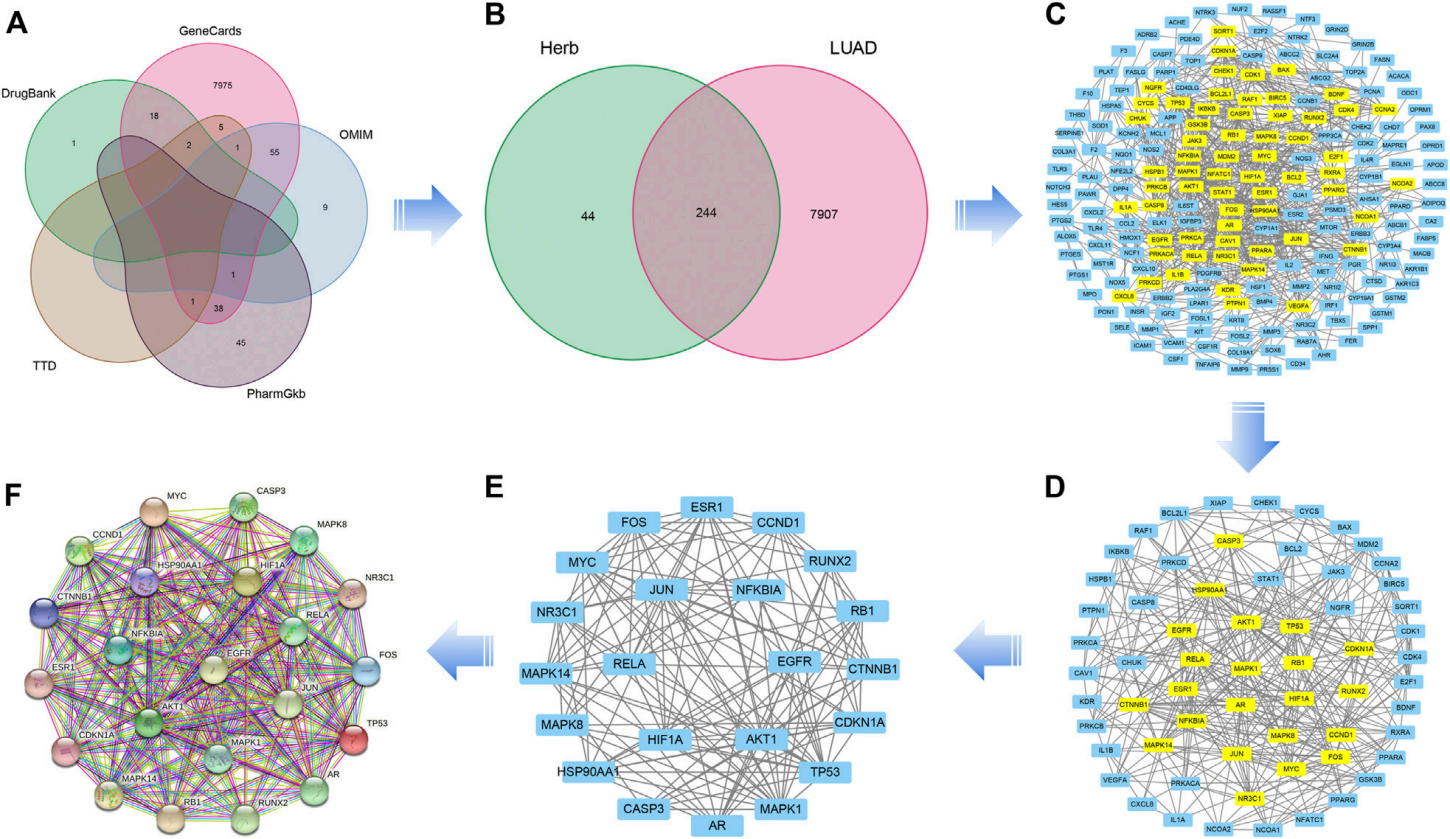
**(A)** A Venn diagram of the intersecting LUAD-related genes constructed using DrugBank, GeneCard, OMIM, PharmGKB, and TTD databases. **(B)** The overlapping genes between QFJDD and LUAD. **(C–E)** The process of identifying twenty two hub genes. **(F)** The PPI network of twenty two hub genes targeted by QFJDD in LUAD. In the PPI diagram, one gene is represented by a circle, and the protein structure is displayed in the center of the circle.

A total of 8277 LUAD-related genes were obtained from a systematic search conducted on DrugBank, GeneCard, OMIM, PharmGKB and TTD databases. After removing the duplicate genes, 8,151 genes were obtained (Figure 3A). Mapping of QFJDD-associated targets and LUAD-related genes generated 244 overlapping genes (Figure 3B). Then, 22 hub genes were identified using the Cytoscape 3.9.1 software (Figures 3C–E). Finally, the obtained hub genes (EGFR, NFKBIA, RELA, MYC, JUN, MAPK1, FOS, CCND1, MAPK14, NR3C1, TP53, HIF1A, CDKN1A, RB1, ESR1, AKT1, AR, CASP3, CTNNB1, RUNX2, MAPK8, and HSP90AA1) were used to construct a PPI network using the STRING database (Figure 3F; Table 4).

**TABLE 4:** Hub genes of QFJDD against LUAD.

UniProt ID	Gene symbol	Gene name	Protein name
P05412	JUN	JUN	Transcription factor AP-1 (Activator protein 1, AP1)
Q04206	RELA	RELA (NFKB3)	Transcription factor p65 (Nuclear factor NF-kappa-B p65 subunit)
P31749	AKT1	AKT1	RAC-alpha serine/threonine-protein kinase
P25963	NFKBIA	NFKBIA (IKBA)	NF-kappa-B inhibitor alpha (I-kappa-B-alpha)
P28482	MAPK1	MAPK1 (ERK2)	Mitogen-activated protein kinase 1 (Extracellular signal-regulated kinase 2, ERK-2)
P01100	FOS	FOS (G0S7)	Proto-oncogene c-Fos (Cellular oncogene fos) (G0/G1 switch regulatory protein 7)
Q16539	MAPK14	MAPK14	Mitogen-activated protein kinase 14
Q16665	HIF1A	HIF1A	Hypoxia-inducible factor 1-alpha
P00533	EGFR	EGFR (ERBB)	Epidermal growth factor receptor
P10275	AR	AR (NR3C4)	Androgen receptor
P42574	CASP3	CASP3 (CPP32)	Caspase-3
P35222	CTNNB1	CTNNB1 (CTNNB)	Catenin beta-1
Q13950	RUNX2	RUNX2 (AML3)	Runt-related transcription factor 2
P45983	MAPK8	MAPK8 (JNK1)	Mitogen-activated protein kinase 8
P01106	MYC	MYC	Myc proto-oncogene protein
P24385	CCND1	CCND1 (BCL1)	G1/S-specific cyclin-D1 (B-cell lymphoma 1 protein, BCL-1)
P04150	NR3C1	NR3C1 (GRL)	Glucocorticoid receptor, GR (Nuclear receptor subfamily 3 group C member 1)
P04637	TP53	TP53 (P53)	Cellular tumor antigen p53 (Antigen NY-CO-13) (Phosphoprotein p53) (Tumor suppressor p53)
P38936	CDKN1A	CDKN1A	Cyclin-dependent kinase inhibitor 1 (CDK-interacting protein 1)
P06400	RB1	RB1	Retinoblastoma-associated protein (p105-Rb) (p110-RB1) (pRb, Rb) (pp110)
P03372	ESR1	ESR1	Estrogen receptor, ER (ER-alpha)
P07900	HSP90AA1	HSP90AA1	Heat shock protein HSP 90-alpha

### 3.3 The gene ontology and kyoto encyclopedia of genes and genomes pathway enrichment analyses

**FIGURE 4:**
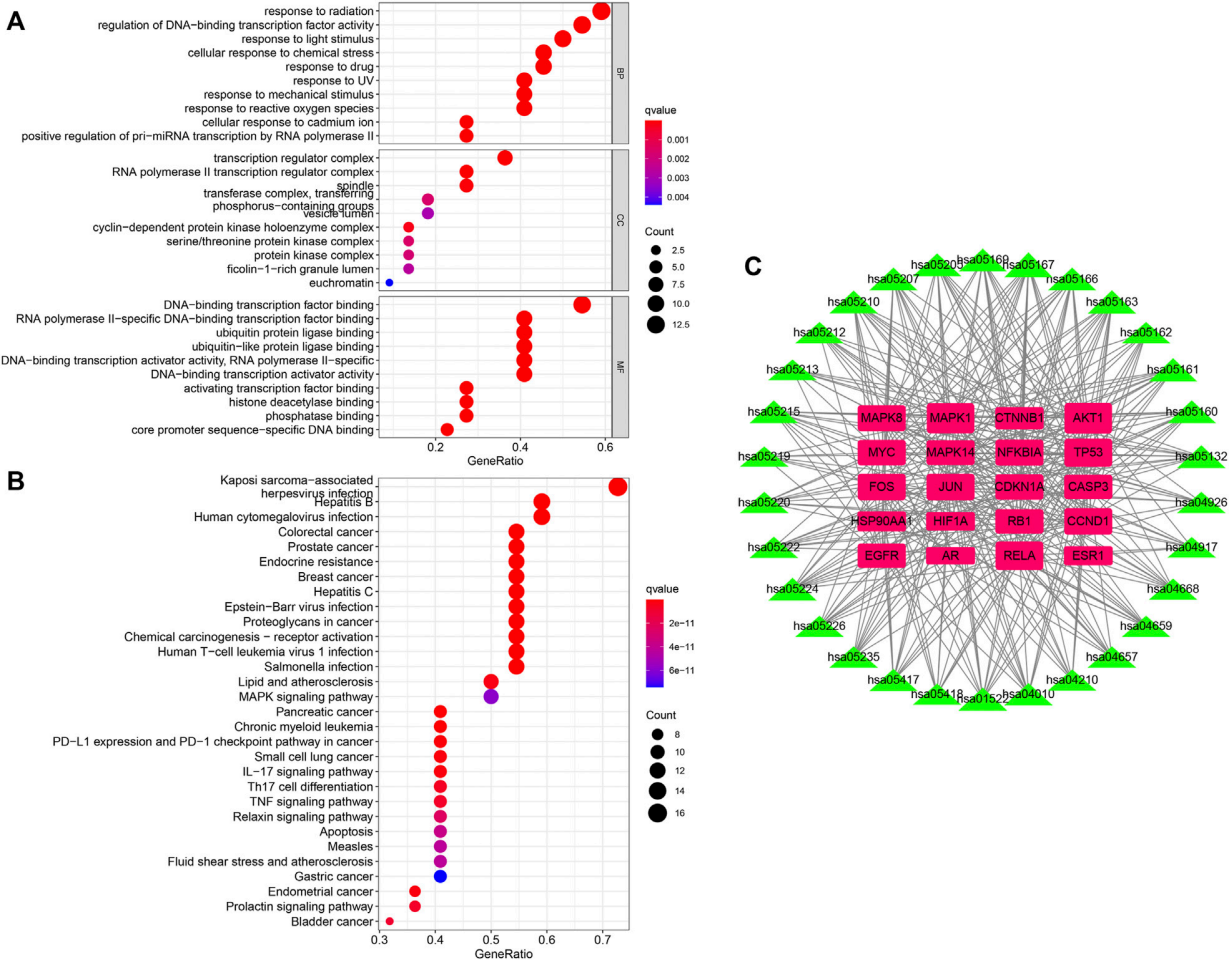
**(A,B)** The GO and KEGG enrichment analyses of hub genes of QFJDD. **(C)** Network of twenty hub genes and thirty signaling pathways. The pink square represents hub gene, and the green triangle indicates signaling pathway. The square and triangle size reflects node degree. The higher the degree value, the bigger the node size.

To investigate the potential functions of the hub genes, GO enrichment analysis of enriched biological processes (BP), cellular components (CC) and molecular functions (MF) was performed (Figure 4A). With regards to BPs, hub genes were enriched in regulation of DNA-binding transcription factor activity, positive regulation of pri-miRNA transcription by RNA polymerase II, response to radiation, cellular response to chemical stress, and response to drug among others. The top five enriched CCs were transcription regulator complex, RNA polymerase II transcription regulator complex, spindle, transferase complex, transferring phosphorus-containing groups and vesicle lumen. The significantly enriched MFs were DNA-binding transcription factor binding, RNA polymerase II-specific DNA-binding transcription factor binding, ubiquitin protein ligase binding, ubiquitin-like protein ligase binding, DNA-binding transcription activator activity, RNA polymerase II-specific and DNA-binding transcription activator activity.

The KEGG analysis of the top 30 signaling pathways revealed that 20 of the 22 hub genes were primarily involved in viral or bacterial infections (Kaposi sarcoma-associated herpesvirus infection, hepatitis B, human T-cell leukemia virus 1 infection, Epstein-Barr virus infection, human cytomegalovirus infection, hepatitis C, Measles, and *salmonella* infection), various cancers (prostate cancer, breast cancer, colorectal cancer, chronic myeloid leukemia, small cell lung cancer, gastric cancer, endometrial cancer, pancreatic cancer, bladder cancer, and proteoglycans in cancer), immune system (PD-L1 expression and PD-1 checkpoint pathway in cancer, Th17 cell differentiation, IL-17 signaling pathway, chemical carcinogenesis-receptor activation, MAPK signaling pathway, apoptosis, and TNF signaling pathway), endocrine system (endocrine resistance, relaxin signaling pathway, and prolactin signaling pathway) and cardiovascular system (lipid and atherosclerosis and fluid shear stress and atherosclerosis) (Figure 4B). Noticeably, the top three genes enriched in the above-mentioned signaling pathways were AKT1, MAPK1, and RELA (Figure 4C, Supplementary Table S7).

### 3.4 The database for annotation, visualization, and integrated discovery pathway analysis

The PD-1/PD-L1 inhibitors are vital in cancer immunotherapy (Pan et al., 2021; Sezer et al., 2021). Based on the results shown in Figures 4B,C, we explored the regulation of tumor cells by the PD-1/PD-L1 pathway.

**FIGURE 5:**
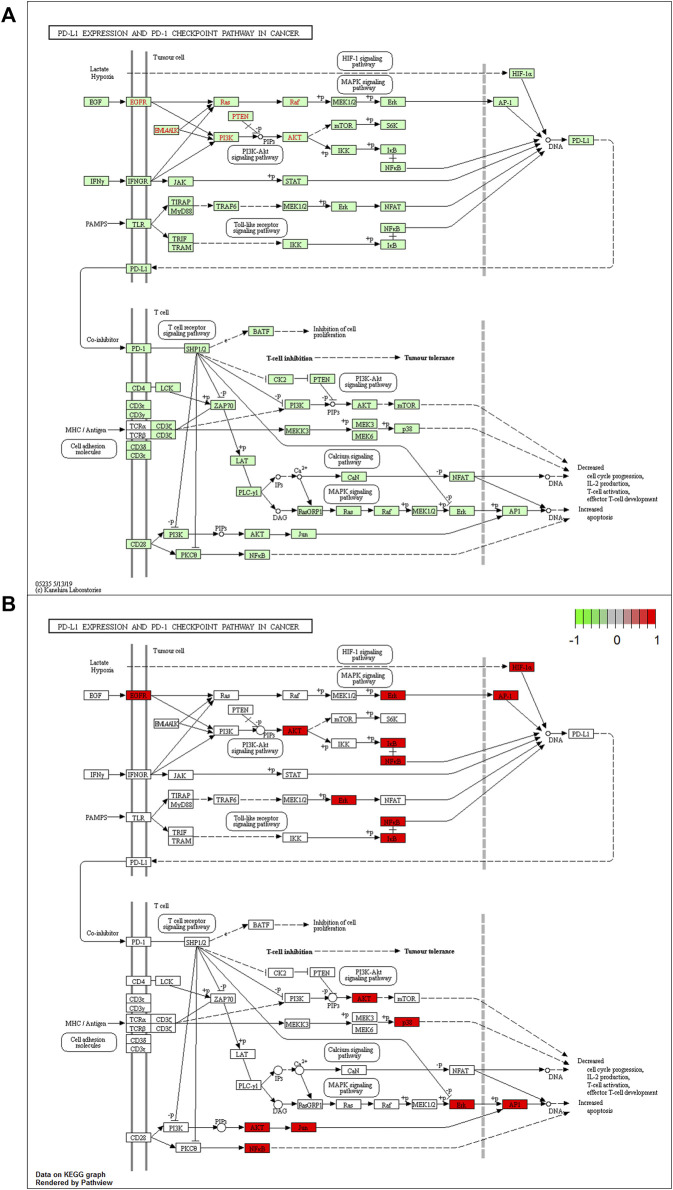
**(A)** The KEGG analysis of PD-L1 expression and PD-1 checkpoint pathway in cancer (hsa05235 pathway). **(B)** Key genes of QFJDD acting on the hsa05235 pathway are labeled in red.

The PD-L1 expression and PD-1 checkpoint pathway in cancer (hsa05235) were drawn in R 4.1.1 software (Figure 5A). The PD-L1 expression in the hsa05235 pathway revealed seven key genes, EGFR, JUN (AP1), RELA (NFKB3), HIF1A, NFKBIA (IKBA), AKT1, and MAPK1 (ERK2), as shown in red (Figure 5B). Notably, JUN, RELA, AKT1, MAPK1, and MAPK14 (MAP kinase p38 alpha) were also involved in T cell functions regulation, including the cell cycle, IL-2 production, T cell activation, effector T-cell development, and apoptosis (Figure 5B).

### 3.5 Molecular docking simulation

**FIGURE 6:**
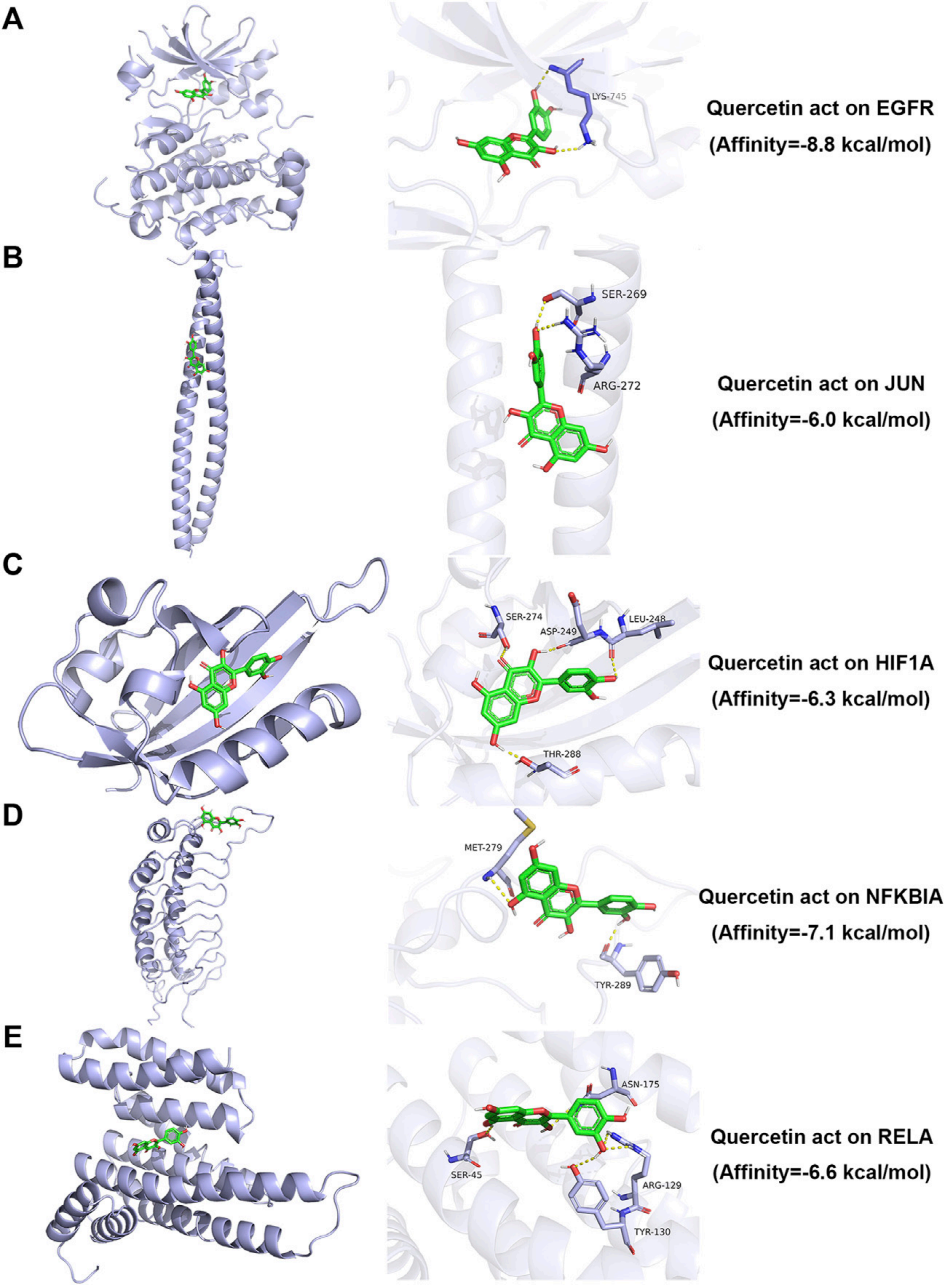
Molecular docking simulation analysis of representative receptor ligand pairs. **(A)** Quercetin act on EGFR. **(B)** Quercetin act on JUN. **(C)** Quercetin act on HIF1A. **(D)** Quercetin act on NFKBIA. **(E)** Quercetin act on RELA.

AutoDock Vina version 1.1.2 software was used to calculate binding active pockets and binding energy between receptor proteins and ligands. Using PyMOL version 2.5, the six core bioactive compounds were observed to enter the active pockets of eight receptors, respectively. The receptor-ligand affinities are shown in Table 5. Three-dimensional structures of several receptor-ligand binding regions are shown in (Figure 6). Taking the HIF1A-quercetin pair in affinity (kcal/mol) as an example for analysis, quercetin formed hydrogen bonds with residues with SER-274, ASP-249, LEU-248, and THR-288 (Figure 6C).

**TABLE 5:** The Affinity (kcal/mol) of molecular docking simulation.

Bioactive compounds	EGFR	JUN	RELA	AKT1	NFKBIA	MAPK1	MAPK14	HIF1A
quercetin	−8.8	−6.0	−6.6	−10.4	−7.0	−7.4	−8.7	−6.3
luteolin	−8.9	−6.1	−6.6	−10.3	−6.7	−7.3	−8.5	−6.7
kaempferol	−8.3	−5.8	−6.6	−9.8	−6.3	−7.2	−8.9	−6.1
wogonin	−8.2	−6.1	−6.5	−9.4	−6.9	−7.3	−8.2	−6.3
baicalein	−8.5	−6.0	−6.5	−9.9	−7.4	−7.4	−9.3	−6.3
acacetin	−8.5	−6.0	−6.6	−9.4	−6.7	−7.1	−9.2	−6.4

### 3.6 Detection of six bioactive compounds in Qingfei Jiedu decoction

**FIGURE 7:**
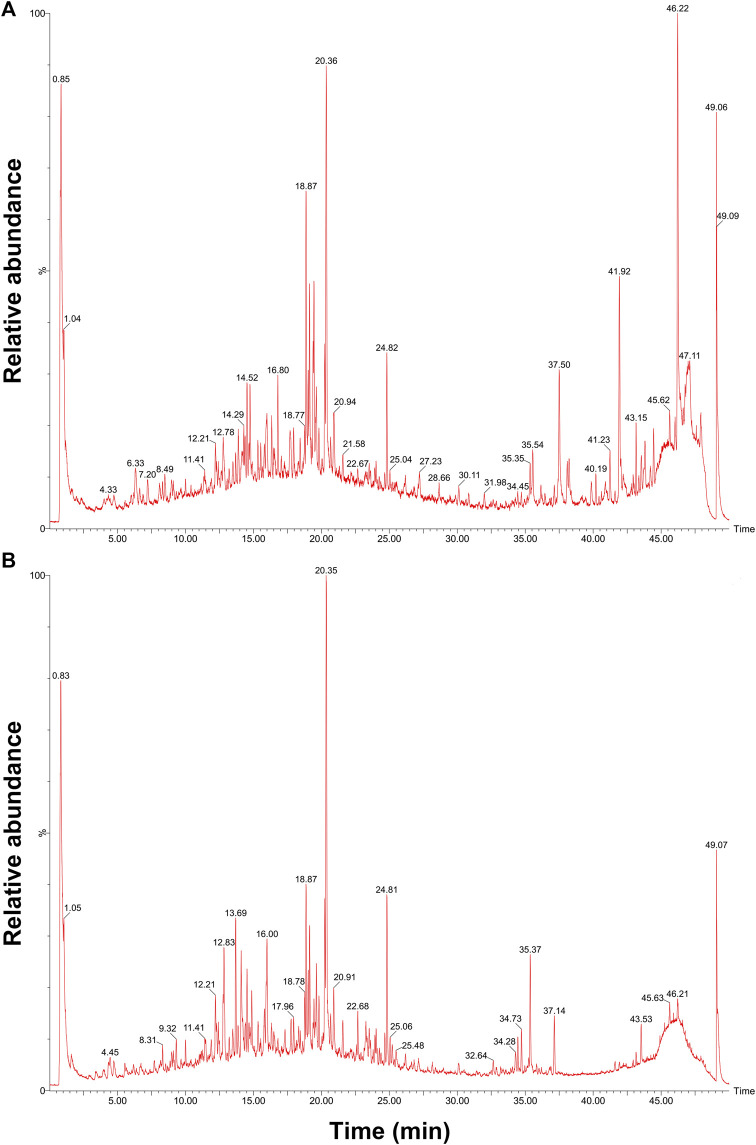
UPLC-Q-TOF-MS chromatogram of QFJDD in **(A)** POS and **(B)** NEG mode. POS, positive; NEG, negative.

Qualitative analysis of QFJDD was carried out using UPLC-Q-TOF-MS (Figures 7A,B). The results showed that quercetin, luteolin, kaempferol, wogonin, baicalein and acacetin in QFJDD were identified by matching to reference standards (Table 6). Quantitative analysis of QFJDD was performed by UPLC (Supplementary Figure S1). The outcomes showed that the contents of quercetin, luteolin, kaempferol, wogonin, baicalein and acacetin in QFJDD were 3.72 × 10^−3^%, 1.45 × 10^−3^%, 8.18 × 10^−4^%, 6.85 × 10^−4^%, 1.82 × 10^−3^% and 3.88 × 10^−4^%, respectively (Supplementary Table S8).

**TABLE 6:** Results of UPLC-Q-TOF-MS analysis of QFJDD.

Compound	Molecular formula	Mode	Retention time (min)	Theoretical m/z	Measured m/z
baicalein	C_15_H_10_O_5_	POS	15.93	271.0606	271.0616
		NEG	15.93	269.0450	269.0413
quercetin	C_15_H_10_O_7_	POS	17.68	303.0505	303.0472
		NEG	17.67	301.0348	301.0319
luteolin	C_15_H_10_O_6_	POS	17.78	287.0556	287.0533
		NEG	17.76	285.0399	285.0406
kaempferol	C_15_H_10_O_6_	POS	20.72	287.0556	287.0533
		NEG	—	—	—
wogonin	C_16_H_12_O_5_	POS	26.10	285.0763	285.0744
		NEG	26.10	283.0606	283.0604
acacetin	C1_6_H_12_O_5_	POS	—	—	—
		NEG	27.04	283.0606	283.0604

POS, positive; NEG, negative.

### 3.7 Effects of Qingfei Jiedu decoction-Containing serum on PD-L1 expression and PD-1 checkpoint pathway in a A549 cell line

**FIGURE 8:**
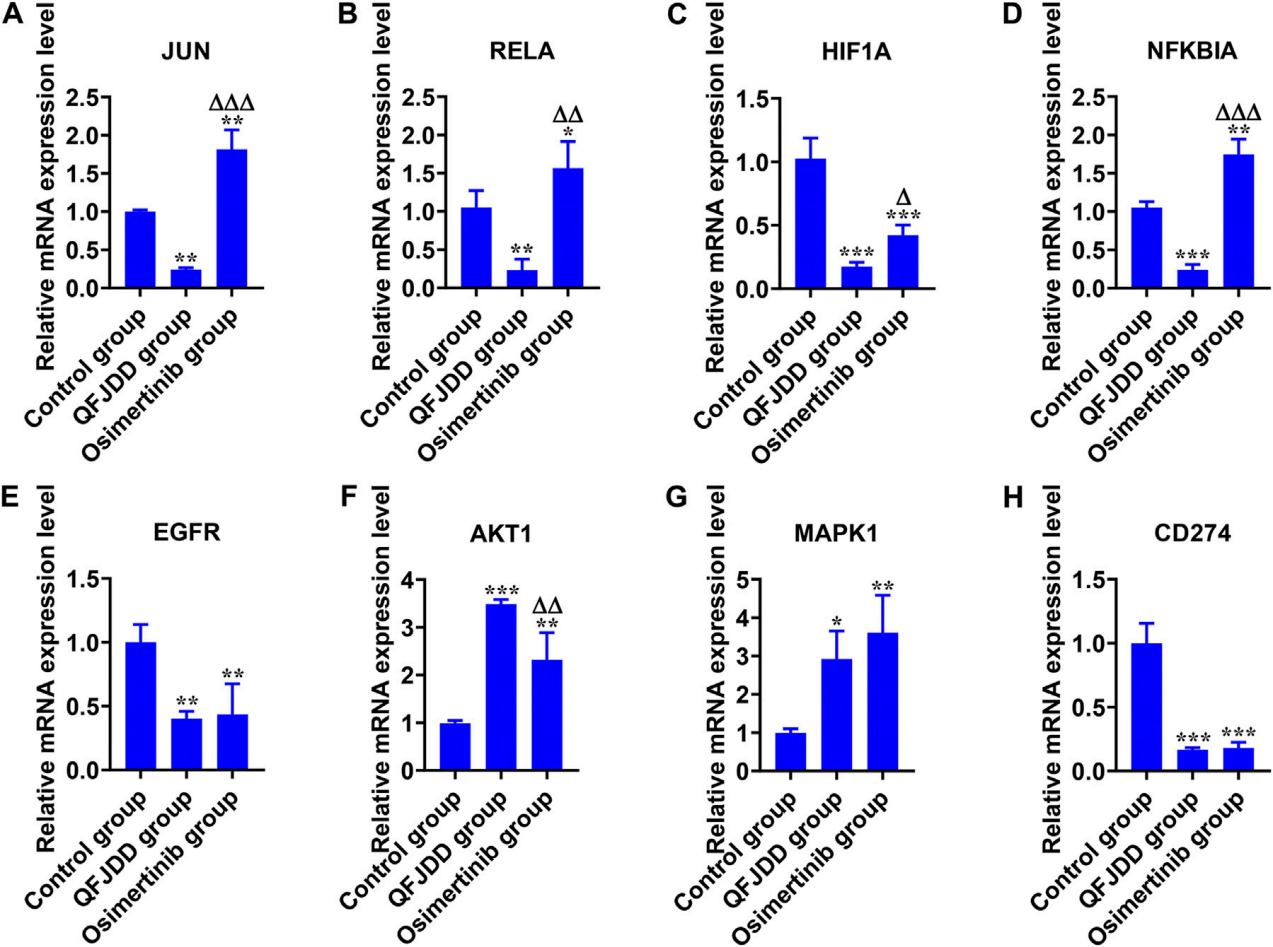
*In vitro* experimental verification. The relative mRNA expression level of **(A)** *JUN*, **(B)** *RELA*, **(C)** *HIF1A*, **(D)** *NFKBIA*, **(E)** *EGFR*, **(F)** *AKT1*, **(G)** *MAPK1*, and **(H)** *CD274* in A549 cells. *N* = 3. Data are shown as means ± SD. The significance of the results was assessed using the ANOVA. ^*^ *p* < 0.05; ^**^ *p* < 0.01; ^***^ *p* < 0.001 (vs. Control group); ^Δ^ *P*<0.05; ^ΔΔ^ *P*<0.01; ^ΔΔΔ^ *P*<0.001 (vs. QFJDD-containing serum group).

Cellular experiments were conducted to validate the effects of QFJDD-containing serum on key genes of the hsa05235 pathway. mRNA expressions of EGFR, HIF1A and CD274 in QFJDD-containing serum group and Osimertinib group were significantly reduced relative to control group (*p* < 0.01; *p* < 0.001, Figures 8C,E,H). Transcription levels of JUN and RELA (*p* < 0.01, Figures 8A,B) as well as NFKBIA (*p* < 0.001, Figure 8D) were significantly decreased in the QFJDD-containing group relative to the control group. On the contrary, expression of JUN and NFKBIA (*p* < 0.001, Figures 8A,D) as well as RELA (*p* < 0.01, Figure 8B) were markedly increased in the Osimertinib group relative to QFJDD-containing serum group. Moreover, compared with the control group, the mRNA expression of AKT1 (*p* < 0.001, Figure 8F) and MAPK1 (*p* < 0.05, Figure 8G **)** in the QFJDD-containing serum group and Osimertinib group was significantly increased. In conclusion, QFJDD-containing serum inhibits PD-L1 expression by regulating the above key genes.

### 3.8 Effects of Qingfei Jiedu decoction on PD-L1 expression in tumor tissues and CD8^+^PD-1^+^% in spleen tissues from Lewis lung cancer-bearing mice

**FIGURE 9:**
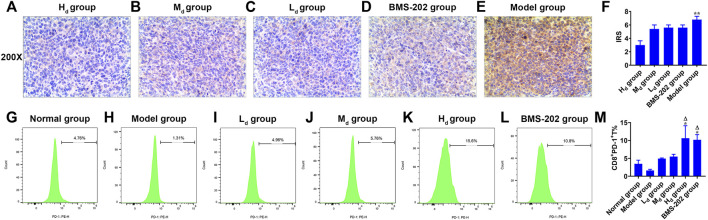
*In vivo* experimental validation. **(A–E)** The representative images (200×) of PD-L1 IHC staining of Lewis lung cancer tissues in each group. **(F)** Intergroup comparison of PD-L1 IRS in LLC tissues. *N* = 5. Data are means ± SD. Statistical significance was tested with Kruskal-Wallis nonparametric analysis of variance. ^**^ *p* < 0.01 (vs. H_d_ group). **(G–M)** Comparison of CD8^+^PD-1^+^T% between groups. *N* = 5. Data are presented as means ± SD. Significance was analyzed by the Kruskal-Wallis nonparametric analysis of variance. ^*^ *p* < 0.05 (vs. Normal group); ^Δ^ *P*<0.01 (vs. Model group).

Animal assays to assess the effects of QFJDD on PD-L1 expression levels in LLC tissues (Figures 9A–E) and CD8^+^PD-1^+^% spleen tissues (Figures 9G–L). The results showed that PD-L1 IRS of the H_d_ group was lower than Model group (*p* < 0.01). However, differences between the other groups were insignificant (Figure 9F). Further, analysis of CD8^+^PD-1^+^T percentage in the spleen tissue of the Normal group, Model group, L_d_ group, M_d_ group, H_d_ group, and BMS-202 group by FCM revealed that CD8^+^PD-1^+^T% in H_d_ and BMS-202 groups were significantly elevated relative to Normal or Model groups (*p* < 0.05, *p* < 0.01, Figure 9M). Therefore, QFJDD reduces the PD-L1 IRS in tumor tissues and facilitates the differentiations of CD8^+^T into toxic CD8^+^PD-1^+^T cells.

## 4 Discussion

TCM is safe, feasible and improves the clinical efficacies of chemotherapy, radiotherapy, targeted therapy and immunotherapy (Zhang et al., 2018; Su et al., 2020; Zhang et al., 2021). Therefore, it has been widely used in adjuvant lung cancer treatment. The PD-1/PD-L1 inhibitors are effective for malignant tumor treatment, and have become a focus of immunotherapy (Steven et al., 2016). Primarily, PD-L1 is expressed on tumor cells. This protein inhibits T-cell activation and proliferation, particularly cytotoxic T lymphocytes (CTLs) by binding to PD-1, which is predominantly expressed on activated T cells (Poggio et al., 2019). In healthy cells, PD-1 and PD-L1 regulate T cell response amplitudes and maintains self-tolerance (Ai et al., 2020). However, cancer cells hijack the PD-1/PD-L1 pathway to evade immune surveillance by overexpressing PD-L1, resulting in cancer cell proliferation and metastasis. QFJDD suppresses PD-L1 levels in Lewis lung cancer cells and increases the proportions and activities of spleen-derived CD8^+^T cells (Pan et al., 2020b). However, the underlying mechanism of QFJDD was unclear. From an immunoadjuvant therapy perspective, we reveal six core bioactive compounds of QFJDD and eight key genes involved in regulating PD-L1 expression and the proportion of CD8^+^PD-1^+^T cells in LUAD.

HIF1A (hypoxia inducible factor 1, alpha subunit) is a basic helix-loop-helix transcription factor that decreases the survival and cytolytic activities of CD8^+^CTLs, and promotes the expression of immune checkpoint inhibition molecules. In LUAD, PD-L1, a direct target of HIF1A, is positively correlated with HIF1A expression (Chen et al., 2020). Blocking HIF1A and PD-L1 enhance T cell activities (Noman et al., 2014). UPLC-Q-TOF-MS and molecular docking revealed that bioactive components of QFJDD acting on HIF1A were quercetin, luteolin, kaempferol, wogonin, baicalein and acacetin (Table 5, Table 6). According to previous studies, quercetin, luteolin and baicalein inhibit the expression of PD-L1 and restore the destruction of tumor cells by T cells (Ke et al., 2019; Jiang et al., 2021; Jing et al., 2021). Moreover, kaempferol significantly inhibits PD-1/PD-L1 interaction (Kim et al., 2020). We found that A549 cells exposed to QFJDD-containing serum had significantly reduced mRNA expressions of HIF1A and CD274 (Figures 8C,H). KEGG analysis showed that HIF1A was a key gene in HIF-1 signaling pathway involved in regulating PD-L1 expression (Figure 5B). These findings suggest that QFJDD suppresses PD-L1 expression in LUAD by inhibiting the HIF-1 signaling pathway and restoring CD8^+^T cell activities.

EGFR (epidermal growth factor receptor) is an important oncogenic signaling pathway in NSCLC. It can directly or indirectly drive PD-L1 overexpression (Li et al., 2018) and affect the abundance of CD8^+^T infiltration in tumor tissues (Zhao et al., 2020). In EGFR mutant NSCLC, activated EGFR induced PD-L1 expression through PI3K/AKT1 and MAPK signaling pathways (Luo et al., 2021), which is in accord with KEGG pathway analysis (Figure 5). In recent years, the inhibitory effects of quercetin, luteolin, kaempferol, wogonin, baicalein and acacetin on EGFR have been reported in literature (Wenzel et al., 2001; Hong et al., 2014; Liu et al., 2016b; Yao et al., 2016; Tian et al., 2021; Ganthala et al., 2022). Furthermore, JUN (jun proto-oncogene, c-Jun) is the key component of dimeric transcription factor AP-1 involved in regulation of PD-L1 expression and activation of CD8^+^T cells (Atsaves et al., 2019; Papavassiliou and Musti, 2020). It is also a target of the MAPK signaling cascade (Sumimoto et al., 2016; Zerdes et al., 2018). According to this study, the QFJDD-containing serum significantly suppressed EGFR, JUN and CD274 mRNA expressions in A549 cells (Figures 8A,E,H). Expressions of MAPK1 in the QFJDD group were significantly high relative to the control group (Figure 8G). Green et al. (2012) reported that the PD-L1 enhancer can bind AP-1 components and increase PD-L1 promoter activities in cHL Reed-Sternberg cells. The IHC assay showed that IRS of PD-L1 in the H_d_ group was lower than in the Model group (Figure 9F). The percentage of CD8^+^PD-1^+^T derived from spleen tissues of LLC bearing mice were apparently increased following treatment with high-dose QFJDD or BMS-202 (Figure 9M). In accordance with previous studies, quercetin, luteolin, kaempferol, wogonin, baicalein, and acacetin inhibit the activation of c-Jun N-terminal kinase and attenuate the activation of the AP-1 transcription factor (Jang et al., 2008; Chen et al., 2008; Fong et al., 2010; Chen et al., 2012; Kim et al., 2012; Chen et al., 2013; Wang et al., 2014). Furthermore, kaempferol blocks the interactions between PD-1 and PD-L1 (Kim et al., 2020) whereas wogonin can down-regulate mRNA expression of ERK2 in melanoma cells (Chen et al., 2017). Thus, QFJDD suppresses PD-L1 expression in LUAD by inhibiting mRNA transcriptions of EGFR and JUN, and increasing the proportions of CD8^+^PD-1^+^T.

NF-κB (Nuclear factor kappa-light-chain-enhancer of activated B cells) is synthesized in the cytoplasm and binds to IκB to form an inactive complex (Zhang et al., 2017a). The heterodimer p50/p65 is a classic representative of the NF-κB family encoded by NFKB1 and RELA genes, respectively. IκBα is encoded by NFKBIA and is phosphorylated and degraded by IκB kinase (IKK), leading to activation of free p-p50/p65 and nuclear translocation (Antonangeli et al., 2020). The *in vitro* cell assay revealed that following treatment with QFJDD-containing serum, mRNA expressions of RELA and NFKBIA in A549 cells were suppressed while AKT1 levels were increased (Figures 8B,D,F). Quercetin, luteolin, kaempferol, wogonin, baicalein, and acacetin have been shown to suppress the NF-κB pathway (Chen et al., 2012; Kim et al., 2014; Sikder et al., 2014; Li et al., 2016; Tuorkey, 2016; Zhang et al., 2017b), consistent with molecular docking and qRT-PCR outcomes. The KEGG pathway analysis showed that NFκB is associated with PD-L1, and was also related to T cells apoptosis (Figure 5B). The PI3K/Akt/mTOR signaling pathway has been shown to affect immunity by regulating PD-L1 expression. Besides, IKK can be activated by AKT1 (Zhao et al., 2019). The NF-κB signal is also associated with PD-L1 and CD8^+^T (Antonangeli et al., 2020). These results suggest that QFJDD downregulates mRNA expression level of PD-L1 by suppressing p65 synthesis and inhibiting IκBα phosphorylation. In addition, the increase in CD8^+^PD-1^+^T% was also related to inhibition of the NFκB signaling pathway by QFJDD.

## 5 Conclusion

This study identified six core bioactive compounds in QFJDD. Then, eight key genes, including EGFR, JUN, RELA, HIF1A, NFKBIA, MAPK1, AKT1, and MAPK14, were identified by mapping the bioactive compounds of QFJDD to the targets of hsa05235 pathway. Meanwhile, EGFR, HIF-1, JUN, and NFκB signaling pathways were shown to be involved in regulating PD-L1 expression and CD8^+^PD-1^+^T% in LUAD. Moreover, the therapeutic potential of QFJDD holds tremendous promise for five areas, including virus, cancer, immunity, endocrine system, and cardiovascular system. Our findings provide a scientific basis for clinical applications of QFJDD in LUAD treatment.

## Data Availability

The original contributions presented in the study are included in the article/Supplementary Materials, further inquiries can be directed to the corresponding author.

## References

[B1] Ai L. Xu A. Xu J. (2020). Roles of PD-1/PD-L1 pathway: Signaling, cancer, and beyond. Adv. Exp. Med. Biol. 1248, 33–59. 10.1007/978-981-15-3266-5_3 32185706

[B2] Amberger J. S. Hamosh A. (2017). Searching online mendelian inheritance in man (OMIM): A knowledgebase of human genes and genetic phenotypes. Curr. Protoc. Bioinforma. 58, 1. 10.1002/cpbi.27 PMC5662200 28654725

[B3] Antonangeli F. Natalini A. Garassino M. C. Sica A. Santoni A. Di Rosa F. (2020). Regulation of PD-L1 expression by NF-κB in cancer. Front. Immunol. 11, 584626. 10.3389/fimmu.2020.584626 33324403 PMC7724774

[B4] Atsaves V. Leventaki V. Rassidakis G. Z. Claret F. X. (2019). AP-1 transcription factors as regulators of immune responses in cancer. Cancers (Basel) 11 (7), E1037. 10.3390/cancers11071037 31340499 PMC6678392

[B5] Bewick V. Cheek L. Ball J. (2004). Statistics review 9: One-way analysis of variance. Crit. Care 8 (2), 130–136. 10.1186/cc2836 15025774 PMC420045

[B6] Chen B. Li L. Li M. Wang X. (2020). HIF1A expression correlates with increased tumor immune and stromal signatures and aggressive phenotypes in human cancers. Cell. Oncol. 43 (5), 877–888. 10.1007/s13402-020-00534-4 PMC12990741 32488852

[B7] Chen H. J. Lin C. M. Lee C. Y. Shih N. C. Peng S. F. Tsuzuki M. (2013). Kaempferol suppresses cell metastasis via inhibition of the ERK-p38-JNK and AP-1 signaling pathways in U-2 OS human osteosarcoma cells. Oncol. Rep. 30 (2), 925–932. 10.3892/or.2013.2490 23708932

[B8] Chen L. G. Hung L. Y. Tsai K. W. Pan Y. S. Tsai Y. D. Li Y. Z. (2008). Wogonin, a bioactive flavonoid in herbal tea, inhibits inflammatory cyclooxygenase-2 gene expression in human lung epithelial cancer cells. Mol. Nutr. Food Res. 52 (11), 1349–1357. 10.1002/mnfr.200700329 18496814

[B9] Chen P. Shi Q. Xu X. Wang Y. Chen W. Wang H. (2012). Quercetin suppresses NF-κB and MCP-1 expression in a high glucose-induced human mesangial cell proliferation model. Int. J. Mol. Med. 30 (1), 119–125. 10.3892/ijmm.2012.955 22469745

[B10] Chen X. Gu T. Wang J. H. Xiong H. Wang Y. Q. Liu G. L. (2017). Effects of wogonin on the mechanism of melanin synthesis in A375 cells. Exp. Ther. Med. 14 (5), 4547–4553. 10.3892/etm.2017.5070 29104663 PMC5658711

[B11] Cyranoski D. (2018). Why Chinese medicine is heading for clinics around the world. Nature 561 (7724), 448–450. 10.1038/d41586-018-06782-7 30258149

[B12] Daina A. Michielin O. Zoete V. (2017). SwissADME: A free web tool to evaluate pharmacokinetics, drug-likeness and medicinal chemistry friendliness of small molecules. Sci. Rep. 7, 42717. 10.1038/srep42717 28256516 PMC5335600

[B13] Daina A. Michielin O. Zoete V. (2019). SwissTargetPrediction: Updated data and new features for efficient prediction of protein targets of small molecules. Nucleic Acids Res. 47 (W1), W357–w364. 10.1093/nar/gkz382 31106366 PMC6602486

[B14] DennisG.Jr.ShermanB. T.HosackD. A.YangJ.GaoW.LaneH. C. (2003). David: Database for annotation, visualization, and integrated discovery. Genome Biol. 4 (5), R60. 10.1186/gb-2003-4-9-r60 12734009

[B15] Duma N. Santana-Davila R. Molina J. R. (2019). Non-small cell lung cancer: Epidemiology, screening, diagnosis, and treatment. Mayo Clin. Proc. 94 (8), 1623–1640. 10.1016/j.mayocp.2019.01.013 31378236

[B16] Fong Y. Shen K. H. Chiang T. A. Shih Y. W. (2010). Acacetin inhibits TPA-induced MMP-2 and u-PA expressions of human lung cancer cells through inactivating JNK signaling pathway and reducing binding activities of NF-kappaB and AP-1. J. Food Sci. 75 (1), H30–H38. 10.1111/j.1750-3841.2009.01438.x 20492175

[B17] Ganthala P. D. Alavala S. Chella N. Andugulapati S. B. Bathini N. B. Sistla R. (2022). Co-encapsulated nanoparticles of Erlotinib and Quercetin for targeting lung cancer through nuclear EGFR and PI3K/AKT inhibition. Colloids Surf. B Biointerfaces 211, 112305. 10.1016/j.colsurfb.2021.112305 34998178

[B18] Gaudet P. Logie C. Lovering R. C. Kuiper M. Lægreid A. Thomas P. D. (2021). Gene Ontology representation for transcription factor functions. Biochim. Biophys. Acta. Gene Regul. Mech. 1864 (11-12), 194752. 10.1016/j.bbagrm.2021.194752 34461313

[B19] Gong L. Whirl-Carrillo M. Klein T. E. (2021). PharmGKB, an integrated resource of pharmacogenomic knowledge. Curr. Protoc. 1 (8), e226. 10.1002/cpz1.226 34387941 PMC8650697

[B20] Green M. R. Rodig S. Juszczynski P. Ouyang J. Sinha P. O'Donnell E. (2012). Constitutive AP-1 activity and EBV infection induce PD-L1 in hodgkin lymphomas and posttransplant lymphoproliferative disorders: Implications for targeted therapy. Clin. Cancer Res. 18 (6), 1611–1618. 10.1158/1078-0432.Ccr-11-1942 22271878 PMC3321508

[B21] Hong Z. Cao X. Li N. Zhang Y. Lan L. Zhou Y. (2014). Luteolin is effective in the non-small cell lung cancer model with L858R/T790M EGF receptor mutation and erlotinib resistance. Br. J. Pharmacol. 171 (11), 2842–2853. 10.1111/bph.12610 24471765 PMC4243859

[B22] Jain P. Jain C. Velcheti V. (2018). Role of immune-checkpoint inhibitors in lung cancer. Ther. Adv. Respir. Dis. 12, 1. 10.1177/1753465817750075 PMC5937156 29385894

[B23] Jang S. Kelley K. W. Johnson R. W. (2008). Luteolin reduces IL-6 production in microglia by inhibiting JNK phosphorylation and activation of AP-1. Proc. Natl. Acad. Sci. U. S. A. 105 (21), 7534–7539. 10.1073/pnas.0802865105 18490655 PMC2396685

[B24] Jiang Z. B. Wang W. J. Xu C. Xie Y. J. Wang X. R. Zhang Y. Z. (2021). Luteolin and its derivative apigenin suppress the inducible PD-L1 expression to improve anti-tumor immunity in KRAS-mutant lung cancer. Cancer Lett. 515, 36–48. 10.1016/j.canlet.2021.05.019 34052328

[B25] Jing L. Lin J. Yang Y. Tao L. Li Y. Liu Z. (2021). Quercetin inhibiting the PD-1/PD-L1 interaction for immune-enhancing cancer chemopreventive agent. Phytother. Res. 35 (11), 6441–6451. 10.1002/ptr.7297 34560814

[B26] Kanehisa M. Furumichi M. Tanabe M. Sato Y. Morishima K. (2017). Kegg: New perspectives on genomes, pathways, diseases and drugs. Nucleic Acids Res. 45 (D1), D353–D361. 10.1093/nar/gkw1092 27899662 PMC5210567

[B27] Ke M. Zhang Z. Xu B. Zhao S. Ding Y. Wu X. (2019). Baicalein and baicalin promote antitumor immunity by suppressing PD-L1 expression in hepatocellular carcinoma cells. Int. Immunopharmacol. 75, 105824. 10.1016/j.intimp.2019.105824 31437792

[B28] Kim H. R. Park C. G. Jung J. Y. (2014). Acacetin (5, 7-dihydroxy-4'-methoxyflavone) exhibits *in vitro* and *in vivo* anticancer activity through the suppression of NF-κB/Akt signaling in prostate cancer cells. Int. J. Mol. Med. 33 (2), 317–324. 10.3892/ijmm.2013.1571 24285354

[B29] Kim J. H. Kim Y. S. Choi J. G. Li W. Lee E. J. Park J. W. (2020). Kaempferol and its glycoside, kaempferol 7-O-rhamnoside, inhibit PD-1/PD-L1 interaction *in vitro* . Int. J. Mol. Sci. 21 (9), E3239. 10.3390/ijms21093239 32375257 PMC7247329

[B30] Kim K. C. Kang S. S. Lee J. Park D. Hyun J. W. (2012). Baicalein attenuates oxidative stress-induced expression of matrix metalloproteinase-1 by regulating the ERK/JNK/AP-1 pathway in human keratinocytes. Biomol. Ther. 20 (1), 57–61. 10.4062/biomolther.2012.20.1.057 PMC3792202 24116275

[B31] Kim S. Chen J. Cheng T. Gindulyte A. He J. He S. (2021). PubChem in 2021: New data content and improved web interfaces. Nucleic Acids Res. 49 (D1), D1388–d1395. 10.1093/nar/gkaa971 33151290 PMC7778930

[B32] Lee J. H. Lee J. H. Ahn B. K. Paik S. S. Lee K. H. (2020). Prognostic value of B-cell linker protein in colorectal cancer. Pathol. Res. Pract. 216 (3), 152821. 10.1016/j.prp.2020.152821 31980295

[B33] Li J. Ma J. Wang K. S. Mi C. Wang Z. Piao L. X. (2016). Baicalein inhibits TNF-α-induced NF-κB activation and expression of NF-κB-regulated target gene products. Oncol. Rep. 36 (5), 2771–2776. 10.3892/or.2016.5108 27667548

[B34] Li X. B. Niu C. L. Chen W. Y. Chen Y. Li Z. Z. (2020). Effect of danggui-shaoyao-san-containing serum on the renal tubular epithelial-mesenchymal transition of diabetic nephropathy. Curr. Pharm. Biotechnol. 21 (12), 1204–1212. 10.2174/1389201021666200416094318 32297575

[B35] Li X. Lian Z. Wang S. Xing L. Yu J. (2018). Interactions between EGFR and PD-1/PD-L1 pathway: Implications for treatment of NSCLC. Cancer Lett. 418, 1–9. 10.1016/j.canlet.2018.01.005 29309815

[B36] Liu X. Tian S. Liu M. Jian L. Zhao L. (2016b). Wogonin inhibits the proliferation and invasion, and induces the apoptosis of HepG2 and Bel7402 HCC cells through NF-κB/Bcl-2, EGFR and EGFR downstream ERK/AKT signaling. Int. J. Mol. Med. 38 (4), 1250–1256. 10.3892/ijmm.2016.2700 27499272

[B37] Liu Z. Guo F. Wang Y. Li C. Zhang X. Li H. (2016a). BATMAN-TCM: A Bioinformatics analysis tool for molecular mechANism of traditional Chinese medicine. Sci. Rep. 6, 21146. 10.1038/srep21146 26879404 PMC4754750

[B38] Luo Y. Ma S. Sun Y. Peng S. Zeng Z. Han L. (2021). MUC3A induces PD-L1 and reduces tyrosine kinase inhibitors effects in EGFR-mutant non-small cell lung cancer. Int. J. Biol. Sci. 17 (7), 1671–1681. 10.7150/ijbs.57964 33994852 PMC8120466

[B39] Nair A. B. Jacob S. (2016). A simple practice guide for dose conversion between animals and human. J. Basic Clin. Pharm. 7 (2), 27–31. 10.4103/0976-0105.177703 27057123 PMC4804402

[B40] Noman M. Z. Desantis G. Janji B. Hasmim M. Karray S. Dessen P. (2014). PD-L1 is a novel direct target of HIF-1α, and its blockade under hypoxia enhanced MDSC-mediated T cell activation. J. Exp. Med. 211 (5), 781–790. 10.1084/jem.20131916 24778419 PMC4010891

[B41] Pan J. Chen F. Yang H. Tang J. Guan C. Shi Y. (2020b). Effects of Qingfei Jiedu decoction on the growth of subcutaneous grafts of Lewis lung cancer in mice and immunosuppression of tumor microenvironment. China J. Traditional Chin. Med. Pharm. 35 (11), 5752–5755.

[B42] Pan J. Yang H. Zhu L. Lou Y. (2021). Correlation of ARNTL2 with immune infiltration and its role as a potential prognostic biomarker in lung adenocarcinoma. Clin. Complementary Med. Pharmacol. 1 (1), 100005. 10.1016/j.ccmp.2021.100005

[B43] Pan J. Zhu L. Song K. Yang H. Lou Y. Shi Y. (2020a). Exploration medication rule of song kang applying traditional Chinese medicine to assist gefitinib in syndrome differentiation and treatment of lung adenocarcinoma based on R language. J. Zhejiang Chin. Med. Univ. 44 (05), 419–425. 10.16466/j.issn1005-5509.2020.05.003

[B44] Papavassiliou A. G. Musti A. M. (2020). The multifaceted output of c-jun biological activity: Focus at the junction of CD8 T cell activation and exhaustion. Cells 9 (11), E2470. 10.3390/cells9112470 33202877 PMC7697663

[B45] Poggio M. Hu T. Pai C. C. Chu B. Belair C. D. Chang A. (2019). Suppression of exosomal PD-L1 induces systemic anti-tumor immunity and memory. Cell 177 (2), 414–427. e413. 10.1016/j.cell.2019.02.016 30951669 PMC6499401

[B46] Ru J. Li P. Wang J. Zhou W. Li B. Huang C. (2014). Tcmsp: A database of systems pharmacology for drug discovery from herbal medicines. J. Cheminform. 6, 13. 10.1186/1758-2946-6-13 24735618 PMC4001360

[B47] Seeliger D. de Groot B. L. (2010). Ligand docking and binding site analysis with PyMOL and Autodock/Vina. J. Comput. Aided. Mol. Des. 24 (5), 417–422. 10.1007/s10822-010-9352-6 20401516 PMC2881210

[B48] Sezer A. Kilickap S. Gümüş M. Bondarenko I. Özgüroğlu M. Gogishvili M. (2021). Cemiplimab monotherapy for first-line treatment of advanced non-small-cell lung cancer with PD-L1 of at least 50%: A multicentre, open-label, global, phase 3, randomised, controlled trial. Lancet 397 (10274), 592–604. 10.1016/s0140-6736(21)00228-2 33581821

[B49] Shannon P. Markiel A. Ozier O. Baliga N. S. Wang J. T. Ramage D. (2003). Cytoscape: A software environment for integrated models of biomolecular interaction networks. Genome Res. 13 (11), 2498–2504. 10.1101/gr.1239303 14597658 PMC403769

[B50] Sikder M. A. Lee H. J. Mia M. Z. Park S. H. Ryu J. Kim J. H. (2014). Inhibition of TNF-α-induced MUC5AC mucin gene expression and production by wogonin through the inactivation of NF-κB signaling in airway epithelial cells. Phytother. Res. 28 (1), 62–68. 10.1002/ptr.4954 23463646

[B51] Stelzer G. Rosen N. Plaschkes I. Zimmerman S. Twik M. Fishilevich S. (2016). The GeneCards suite: From gene data mining to disease genome sequence analyses. Curr. Protoc. Bioinforma. 54, 1. 10.1002/cpbi.5 27322403

[B52] Steven A. Fisher S. A. Robinson B. W. (2016). Immunotherapy for lung cancer. Respirology 21 (5), 821–833. 10.1111/resp.12789 27101251

[B53] Su X. L. Wang J. W. Che H. Wang C. F. Jiang H. Lei X. (2020). Clinical application and mechanism of traditional Chinese medicine in treatment of lung cancer. Chin. Med. J. 133 (24), 2987–2997. 10.1097/cm9.0000000000001141 33065603 PMC7752681

[B54] Sumimoto H. Takano A. Teramoto K. Daigo Y. (2016). RAS-Mitogen-Activated protein kinase signal is required for enhanced PD-L1 expression in human lung cancers. PLoS One 11 (11), e0166626. 10.1371/journal.pone.0166626 27846317 PMC5112979

[B55] Sung H. Ferlay J. Siegel R. L. Laversanne M. Soerjomataram I. Jemal A. (2021). Global cancer statistics 2020: GLOBOCAN estimates of incidence and mortality worldwide for 36 cancers in 185 countries. Ca. Cancer J. Clin. 71 (3), 209–249. 10.3322/caac.21660 33538338

[B56] Szklarczyk D. Gable A. L. Nastou K. C. Lyon D. Kirsch R. Pyysalo S. (2021). The STRING database in 2021: Customizable protein-protein networks, and functional characterization of user-uploaded gene/measurement sets. Nucleic Acids Res. 49 (D1), D605–d612. 10.1093/nar/gkaa1074 33237311 PMC7779004

[B57] Theodorsson-Norheim E. (1986). Kruskal-wallis test: BASIC computer program to perform nonparametric one-way analysis of variance and multiple comparisons on ranks of several independent samples. Comput. Methods Programs Biomed. 23 (1), 57–62. 10.1016/0169-2607(86)90081-7 3638187

[B58] Tian J. Li J. Bie B. Sun J. Mu Y. Shi M. (2021). MiR-3663-3p participates in the anti-hepatocellular carcinoma proliferation activity of baicalein by targeting SH3GL1 and negatively regulating EGFR/ERK/NF-κB signaling. Toxicol. Appl. Pharmacol. 420, 115522. 10.1016/j.taap.2021.115522 33838155

[B59] Tu Y. Wu Q. He J. Xu J. Yu S. Wang Q. (2021). Exploring the potential molecular mechanism of scutellaria baicalensis georgi in the treatment of gastric cancer based on network pharmacological analysis and molecular docking Technology. Front. Pharmacol. 12, 697704. 10.3389/fphar.2021.697704 34421596 PMC8378178

[B60] Tuorkey M. J. (2016). Molecular targets of luteolin in cancer. Eur. J. Cancer Prev. 25 (1), 65–76. 10.1097/cej.0000000000000128 25714651 PMC4885545

[B61] Velankar S. Burley S. K. Kurisu G. Hoch J. C. Markley J. L. (2021). The protein Data Bank archive. Methods Mol. Biol. 2305, 3–21. 10.1007/978-1-0716-1406-8_1 33950382

[B62] Wang L. Cheng X. Li H. Qiu F. Yang N. Wang B. (2014). Quercetin reduces oxidative stress and inhibits activation of c-Jun N-terminal kinase/activator protein-1 signaling in an experimental mouse model of abdominal aortic aneurysm. Mol. Med. Rep. 9 (2), 435–442. 10.3892/mmr.2013.1846 24337353 PMC3896506

[B63] Wang Y. Yang H. Chen L. Jafari M. Tang J. (2021). Network-based modeling of herb combinations in traditional Chinese medicine. Brief. Bioinform. 22 (5), bbab106. 10.1093/bib/bbab106 33834186 PMC8425426

[B64] Wang Y. Zhang S. Li F. Zhou Y. Zhang Y. Wang Z. (2020). Therapeutic target database 2020: Enriched resource for facilitating research and early development of targeted therapeutics. Nucleic Acids Res. 48 (D1), D1031–D1041. 10.1093/nar/gkz981 31691823 PMC7145558

[B65] Wenzel U. Kuntz S. Daniel H. (2001). Flavonoids with epidermal growth factor-receptor tyrosine kinase inhibitory activity stimulate PEPT1-mediated cefixime uptake into human intestinal epithelial cells. J. Pharmacol. Exp. Ther. 299 (1), 351–357. 11561098

[B66] Wishart D. S. Feunang Y. D. Guo A. C. Lo E. J. Marcu A. Grant J. R. (2018). DrugBank 5.0: A major update to the DrugBank database for 2018. Nucleic Acids Res. 46 (D1), D1074–D1082. 10.1093/nar/gkx1037 29126136 PMC5753335

[B67] Wu F. Wang L. Zhou C. (2021). Lung cancer in China: Current and prospect. Curr. Opin. Oncol. 33 (1), 40–46. 10.1097/cco.0000000000000703 33165004

[B68] Yao S. Wang X. Li C. Zhao T. Jin H. Fang W. (2016). Kaempferol inhibits cell proliferation and glycolysis in esophagus squamous cell carcinoma via targeting EGFR signaling pathway. Tumour Biol. 37 (8), 10247–10256. 10.1007/s13277-016-4912-6 26831667

[B69] Zerdes I. Matikas A. Bergh J. Rassidakis G. Z. Foukakis T. (2018). Genetic, transcriptional and post-translational regulation of the programmed death protein ligand 1 in cancer: Biology and clinical correlations. Oncogene 37 (34), 4639–4661. 10.1038/s41388-018-0303-3 29765155 PMC6107481

[B70] Zhang L. Zhang F. Y. Li G. F. (2021). Traditional Chinese medicine and lung cancer--From theory to practice. Biomed. Pharmacother. 137, 111381. 10.1016/j.biopha.2021.111381 33601147

[B71] Zhang Q. Lenardo M. J. Baltimore D. (2017a). 30 Years of NF-κB: A blossoming of relevance to human pathobiology. Cell 168 (1-2), 37–57. 10.1016/j.cell.2016.12.012 28086098 PMC5268070

[B72] Zhang R. Ai X. Duan Y. Xue M. He W. Wang C. (2017b). Kaempferol ameliorates H9N2 swine influenza virus-induced acute lung injury by inactivation of TLR4/MyD88-mediated NF-κB and MAPK signaling pathways. Biomed. Pharmacother. 89, 660–672. 10.1016/j.biopha.2017.02.081 28262619

[B73] Zhang X. W. Liu W. Jiang H. L. Mao B. (2018). Chinese herbal medicine for advanced non-small-cell lung cancer: A systematic review and meta-analysis. Am. J. Chin. Med. 46 (5), 923–952. 10.1142/s0192415x18500490 30001642

[B74] Zhao C. Su C. Li X. Zhou C. (2020). Association of CD8 T cell apoptosis and EGFR mutation in non-small lung cancer patients. Thorac. Cancer 11 (8), 2130–2136. 10.1111/1759-7714.13504 32500560 PMC7396381

[B75] Zhao R. Song Y. Wang Y. Huang Y. Li Z. Cui Y. (2019). PD-1/PD-L1 blockade rescue exhausted CD8+ T cells in gastrointestinal stromal tumours via the PI3K/Akt/mTOR signalling pathway. Cell Prolif. 52 (3), e12571. 10.1111/cpr.12571 30714229 PMC6536456

