# Supplementary material for: Qingfei Jiedu decoction inhibits PD-L1 expression in lung adenocarcinoma based on network pharmacology analysis, molecular docking and experimental verification
Source: Front Pharmacol. 2022 Aug 22;13:897966. doi: 10.3389/fphar.2022.897966 (PMC9454399; doi:10.3389/fphar.2022.897966)
Supplement: Supplementary file 1 [file DataSheet1.ZIP › Supplementary Table and Figure/Supplementary Table S1.docx]

**Supplementary Table S1** Mobile phase gradient

| **Time (min)** | **Flow (ml/min)** | **%A** | **%B** | **Curve** |
| --- | --- | --- | --- | --- |
| 0.00 | 0.300 | 5.0 | 95.0 | 0 |
| 2.00 | 0.300 | 5.0 | 95.0 | 6 |
| 32.00 | 0.300 | 45.0 | 55.0 | 6 |
| 47.00 | 0.300 | 100.0 | 0.0 | 6 |
| 48.00 | 0.300 | 100.0 | 0.0 | 6 |
| 48.10 | 0.300 | 5.0 | 95.0 | 6 |
| 50.00 | 0.300 | 5.0 | 95.0 | 6 |
