# Supplementary material for: Qingfei Jiedu decoction inhibits PD-L1 expression in lung adenocarcinoma based on network pharmacology analysis, molecular docking and experimental verification
Source: Front Pharmacol. 2022 Aug 22;13:897966. doi: 10.3389/fphar.2022.897966 (PMC9454399; doi:10.3389/fphar.2022.897966)
Supplement: Supplementary file 1 [file DataSheet1.ZIP › Supplementary Table and Figure/Supplementary Table S3.docx]

**Supplementary Table S3** Gradient elution conditions for the separation of six bioactive compounds in QFJDD

| **Time (min)** | **Flow (ml/min)** | **%A** | **%B** |
| --- | --- | --- | --- |
| 0 | 0.300 | 70 | 30 |
| 1 | 0.300 | 60 | 40 |
| 30 | 0.300 | 40 | 60 |
| 33 | 0.300 | 0 | 100 |
| 35 | 0.300 | 70 | 30 |
| 37 | 0.300 | 70 | 30 |

A, 0.2% phosphoric acid-distilled water; B, methanol.
