# Supplementary material for: Qingfei Jiedu decoction inhibits PD-L1 expression in lung adenocarcinoma based on network pharmacology analysis, molecular docking and experimental verification
Source: Front Pharmacol. 2022 Aug 22;13:897966. doi: 10.3389/fphar.2022.897966 (PMC9454399; doi:10.3389/fphar.2022.897966)
Supplement: Supplementary file 1 [file DataSheet1.ZIP › Supplementary Table and Figure/Supplementary Table S7.docx]

**Supplementary Table 7** The top 30 signaling pathways

| **ID** | **Description** |
| --- | --- |
| hsa05167 | Kaposi sarcoma-associated herpesvirus infection |
| hsa05166 | Human T-cell leukemia virus 1 infection |
| hsa05163 | Human cytomegalovirus infection |
| hsa05161 | Hepatitis B |
| hsa05160 | Hepatitis C |
| hsa05132 | Salmonella infection |
| hsa05162 | Measles |
| hsa04917 | Prolactin signaling pathway |
| hsa04668 | TNF signaling pathway |
| hsa04659 | Th17 cell differentiation |
| hsa04657 | IL-17 signaling pathway |
| hsa04010 | MAPK signaling pathway |
| hsa01522 | Endocrine resistance |
| hsa05417 | Lipid and atherosclerosis |
| hsa05235 | PD-L1 expression and PD-1 checkpoint pathway in cancer |
| hsa05226 | Gastric cancer |
| hsa04215 | Apoptosis |
| hsa05224 | Breast cancer |
| hsa05222 | Small cell lung cancer |
| hsa05220 | Chronic myeloid leukemia |
| hsa05219 | Bladder cancer |
| hsa04926 | relaxin signaling pathway |
| hsa05418 | fluid shear stress and atherosclerosis |
| hsa05215 | Prostate cancer |
| hsa05213 | Endometrial cancer |
| hsa05212 | Pancreatic cancer |
| hsa05210 | Colorectal cancer |
| hsa05207 | Chemical carcinogenesis-receptor activation |
| hsa05205 | Proteoglycans in cancer |
| hsa05169 | Epstein-Barr virus infection |
