# Supplementary material for: Qingfei Jiedu decoction inhibits PD-L1 expression in lung adenocarcinoma based on network pharmacology analysis, molecular docking and experimental verification
Source: Front Pharmacol. 2022 Aug 22;13:897966. doi: 10.3389/fphar.2022.897966 (PMC9454399; doi:10.3389/fphar.2022.897966)
Supplement: Supplementary file 1 [file DataSheet1.ZIP › Supplementary Table and Figure/Supplementary Table S5.docx]

**Supplementary Table S5** Genes and primer sets used for qRT-PCR

| **Gene** | **Forward (5’ to 3’)** | **Reverse (5’ to 3’)** |
| --- | --- | --- |
| JUN | CCAACTCATGCTAACGCAGC | CTCTCCGTCGCAACTTGTCA |
| RELA | ACAGAAGCAGGCTGGAGGTAAGG | GGACAATGCCAGTGCCATACAGG |
| AKT1 | TGACCATGAACGAGTTTGAGTA | GAGGATCTTCATGGCGTAGTAG |
| NFKBIA | GAGACTTTCGAGGAAATACCCC | GTAGCCATGGATAGAGGCTAAG |
| EGFR | CACTTGGGAGCCTGATGTTA | GGTGAGGAACAACCGCTACA |
| MAPK1 | ATGGTGTGCTCTGCTTATGATA | TCTTTCATTTGCTCGATGGTTG |
| HIF1A | CCATTAGAAAGCAGTTCCGCAAGC | GTGGTAGTGGTGGCATTAGCAGTAG |
| CD274 | GCTGCACTAATTGTCTATTGGG | CACAGTAATTCGCTTGTAGTCG |
| GAPDH | GGCATGGGTCAGAAGGATTCC | ATGTCACGCACGATTTCCCGC |
