# Supplementary material for: Qingfei Jiedu decoction inhibits PD-L1 expression in lung adenocarcinoma based on network pharmacology analysis, molecular docking and experimental verification
Source: Front Pharmacol. 2022 Aug 22;13:897966. doi: 10.3389/fphar.2022.897966 (PMC9454399; doi:10.3389/fphar.2022.897966)
Supplement: Supplementary file 1 [file DataSheet1.ZIP › Supplementary Table and Figure/Supplementary Table S8.docx]

**Supplementary Table S8** Content proportion of 6 bioactive compounds in QFJDD

| **Standards** | **Purity of standards** | **Retention time (min)** | **Peak area of sample solution** | **Concentration of sample solution (μg/ml)** | **Content (%)** |
| --- | --- | --- | --- | --- | --- |
| quercetin | 0.9713 | 4.612 | 122488 | 3.859697288 | 3.72×10^-3^ |
| luteolin | 0.9891 | 5.552 | 90611 | 1.480001171 | 1.45×10^-3^ |
| kaempferol | 0.9894 | 7.295 | 37131 | 0.83328775 | 8.18×10^-4^ |
| wogonin | 0.9840 | 9.849 | 38883 | 1.866106326 | 6.85×10^-4^ |
| baicalein | 0.9961 | 13.865 | 12976 | 0.692573462 | 1.82×10^-3^ |
| acacetin | 1.0000 | 16.783 | 16939 | 0.391010679 | 3.88×10^-4^ |
