# Supplementary material for: Qingfei Jiedu decoction inhibits PD-L1 expression in lung adenocarcinoma based on network pharmacology analysis, molecular docking and experimental verification
Source: Front Pharmacol. 2022 Aug 22;13:897966. doi: 10.3389/fphar.2022.897966 (PMC9454399; doi:10.3389/fphar.2022.897966)
Supplement: Supplementary file 1 [file DataSheet1.ZIP › Supplementary Table and Figure/Supplementary Table S4.docx]

**Supplementary Table S4** Standard Curves for Six Reference Standards

| **Reference standards** | **Linear equation** | **R^2^** | **Linear range (μg/ml)** |
| --- | --- | --- | --- |
| quercetin | y=41888X-39187 | 0.9992 | 1.81-48.52 |
| luteolin | y=68304X-10479 | 0.9991 | 1.42-9.93 |
| kaempferol | y=48994X-3695.1 | 0.9992 | 0.46-13.29 |
| wogonin | y=18411X+225.03 | 0.9994 | 0.25-1.73 |
| baicalein | y=28911X-15068 | 0.9994 | 0.63-4.70 |
| acacetin | y=58525X-5944.9 | 0.9993 | 0.40-6.98 |
