# Supplementary material for: Qingfei Jiedu decoction inhibits PD-L1 expression in lung adenocarcinoma based on network pharmacology analysis, molecular docking and experimental verification
Source: Front Pharmacol. 2022 Aug 22;13:897966. doi: 10.3389/fphar.2022.897966 (PMC9454399; doi:10.3389/fphar.2022.897966)
Supplement: Supplementary file 1 [file DataSheet1.ZIP › Supplementary Table and Figure/Supplementary Table S2.docx]

**Supplementary Table S2** Mass parameters

| **MS parameters** | **parameter** |
| --- | --- |
| TOF mass range (m/z) | 50-1200 |
| Sample cone voltage (V) | 40 |
| Source offset voltage (V) | 80 |
| Cone Gas (L/h) | 50 |
| Ion Spray Voltage Floating (kV) | Positive 3.0; Negative 2.5 |
| Ion Source Temperature (°C) | 120 |
| Desolvation temperature (°C) | 500 |
| Desolvation gas flow rate (L/h) | 1000 |
| Nebuliser | 6.0 bar |
