# Supplementary material for: Qingfei Jiedu decoction inhibits PD-L1 expression in lung adenocarcinoma based on network pharmacology analysis, molecular docking and experimental verification
Source: Front Pharmacol. 2022 Aug 22;13:897966. doi: 10.3389/fphar.2022.897966 (PMC9454399; doi:10.3389/fphar.2022.897966)
Supplement: Supplementary file 1 [file DataSheet1.ZIP › Supplementary Table and Figure/Supplementary Table S6.docx]

**Supplementary Table S6** Fifty-two bioactive compounds of QFJDD

| **Molecular ID** | **CID** | **Molecular name** | **2D Structure** |
| --- | --- | --- | --- |
| MOL012266 | 13889022 | rivularin^a^ | 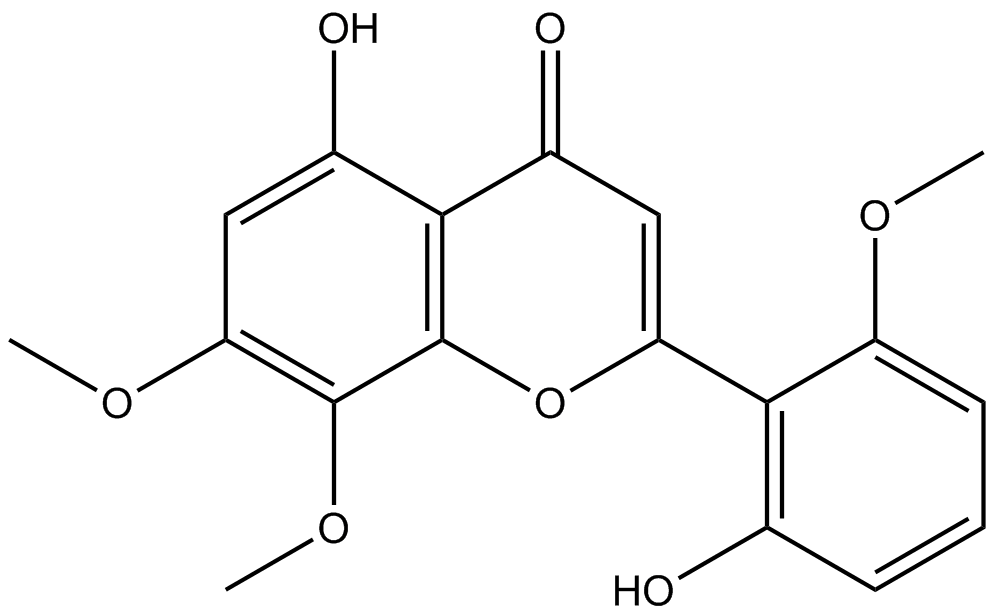 |
| MOL012251 | 5490127 | Chrysin-5-methylether^a^ | 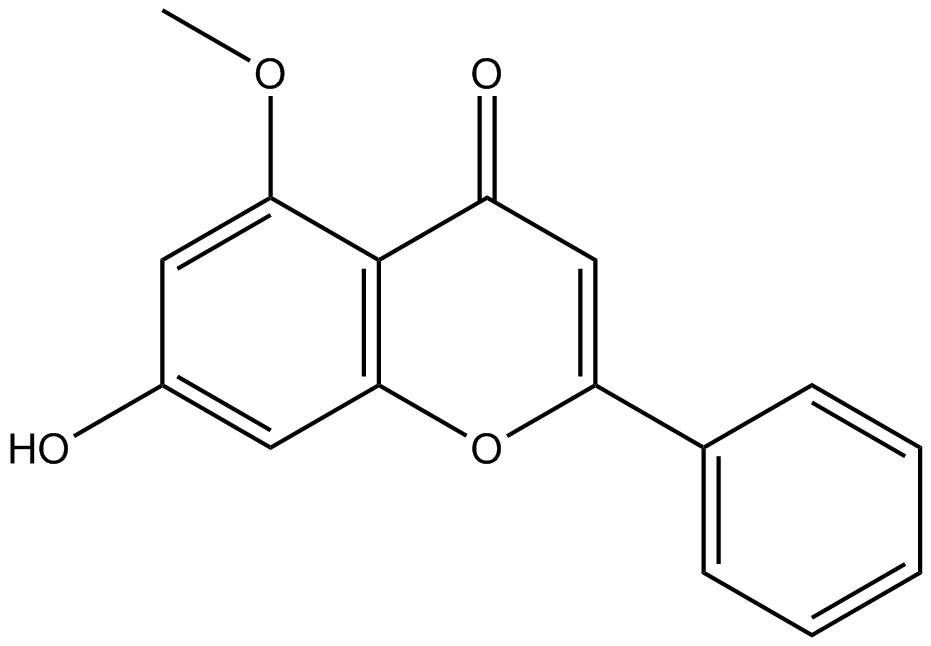 |
| MOL002915 | 161271 | Salvigenin^a^ | 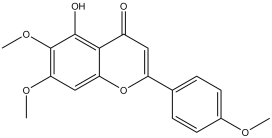 |
| MOL012254 | 173183 | campesterol^a^ | 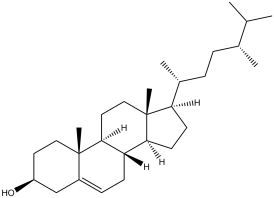 |
| MOL000173 | 5281703 | wogonin^a^ | 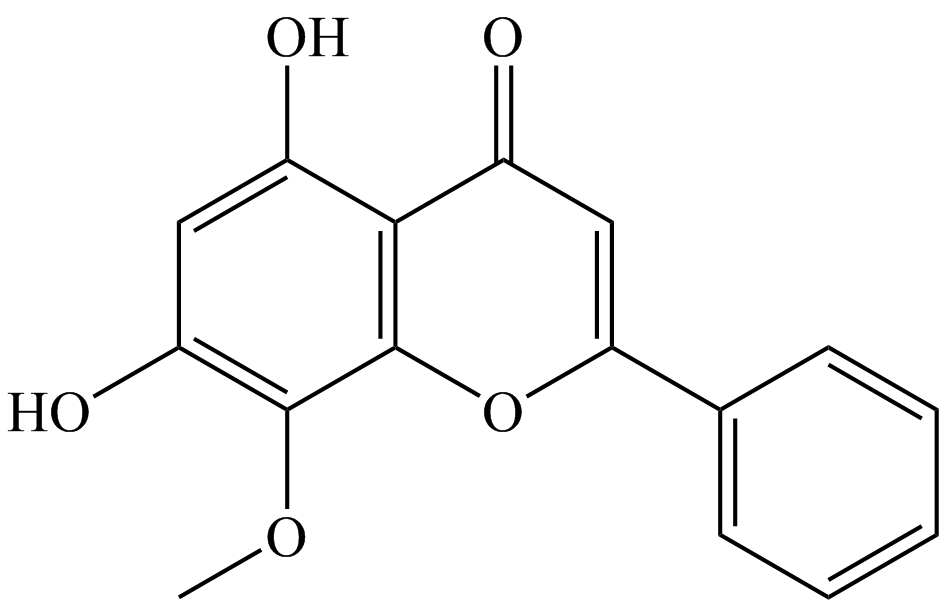 |
| MOL001040 | 667495 | (2R)-5,7-dihydroxy-2-(4-hydroxyphenyl)chroman-4-one^a^ | 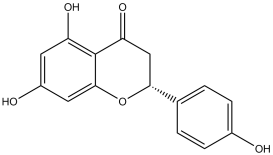 |
| MOL012270 | N/A | Stigmastan-3,5,22-triene^a^ | 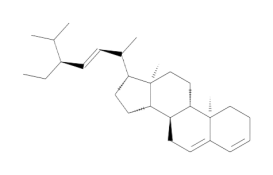 |
| MOL002776 | 64982 | Baicalin^a^ | 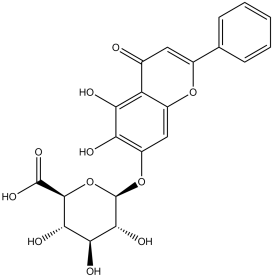 |
| MOL012269 | 129885398 | Stigmasta-5,22-dien-3-ol-acetate^a^ | 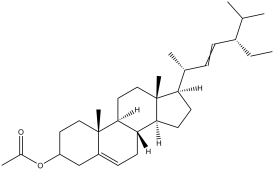 |
| MOL012252 | 129660864 | 9,19-cyclolanost-24-en-3-ol^a^ | 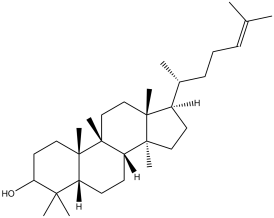 |
| MOL001973 | 5354503 | Sitosteryl acetate^a^ | 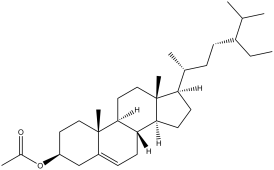 |
| MOL002714 | 5281605 | baicalein^a^ | 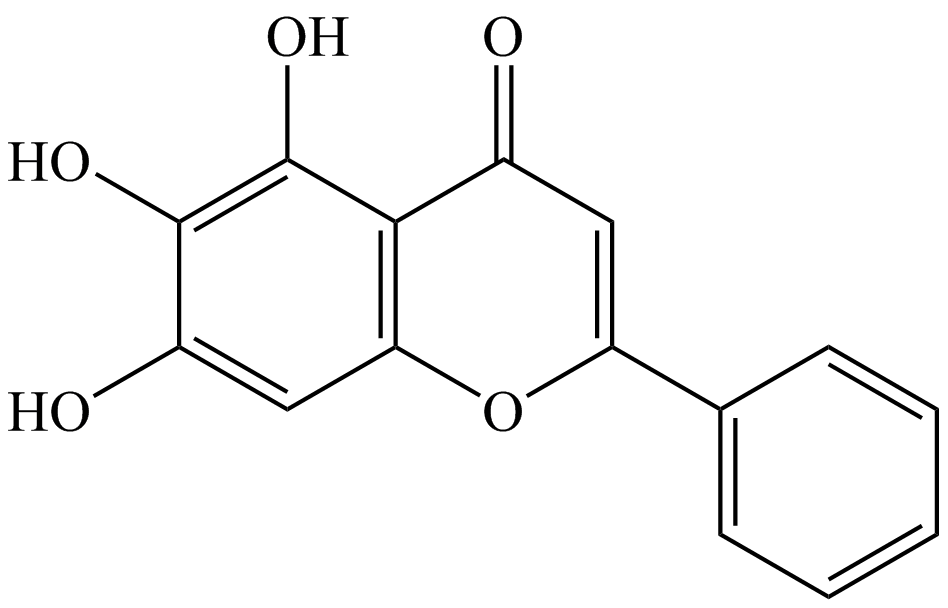 |
| MOL012248 | 14353376 | 5-hydroxy-7,8-dimethoxy-2-(4-methoxyphenyl)chromone^a^ | 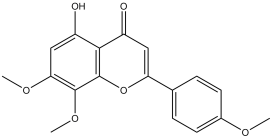 |
| MOL001755 | 15596633 | 24-Ethylcholest-4-en-3-one^a^ | 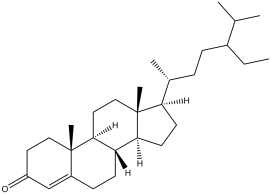 |
| MOL008206 | 188316 | Moslosooflavone^a^ | 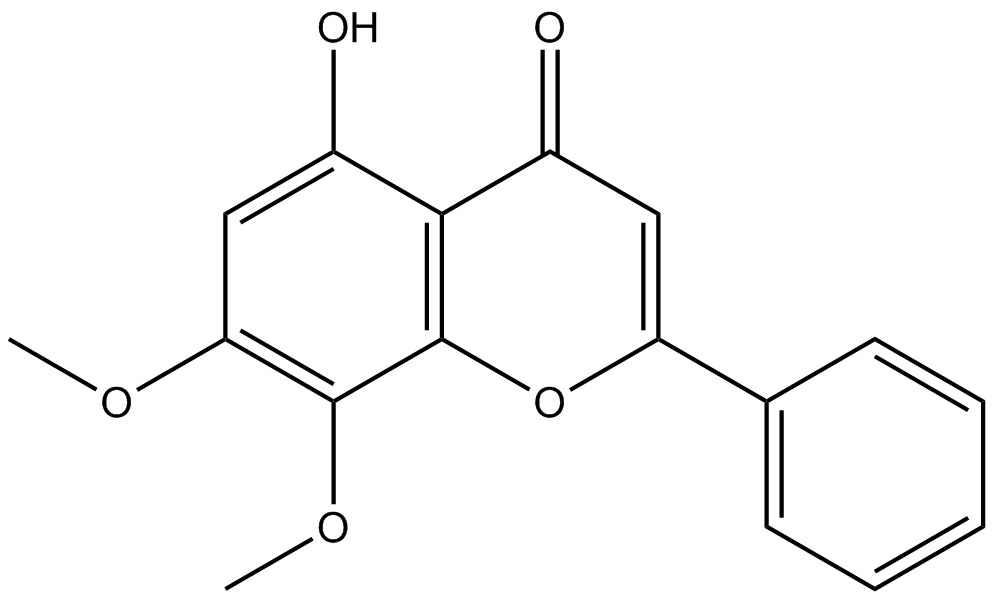 |
| MOL005869 | 5742590 | daucostero_qt^a^ | 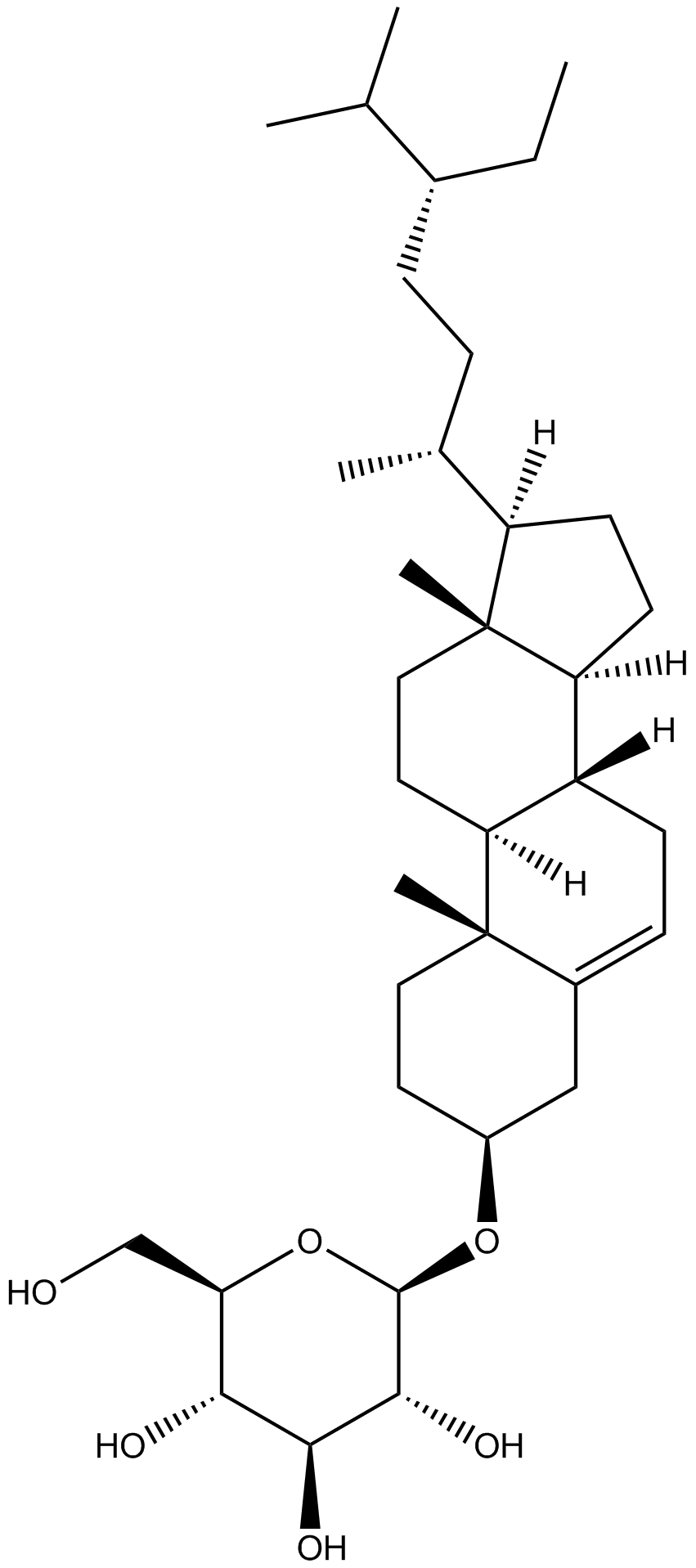 |
| MOL012246 | 42608119 | 5,7,4'-trihydroxy-8-methoxyflavanone^a^ | 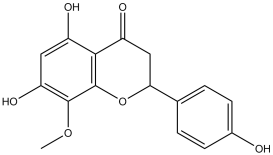 |
| MOL000351 | 5320945 | Rhamnazin^a^ | 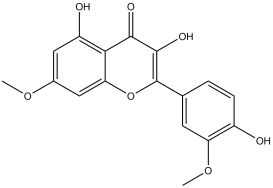 |
| MOL012250 | 14825644 | 7-hydroxy-5,8-dimethoxy-2-phenyl-chromone^a^ | 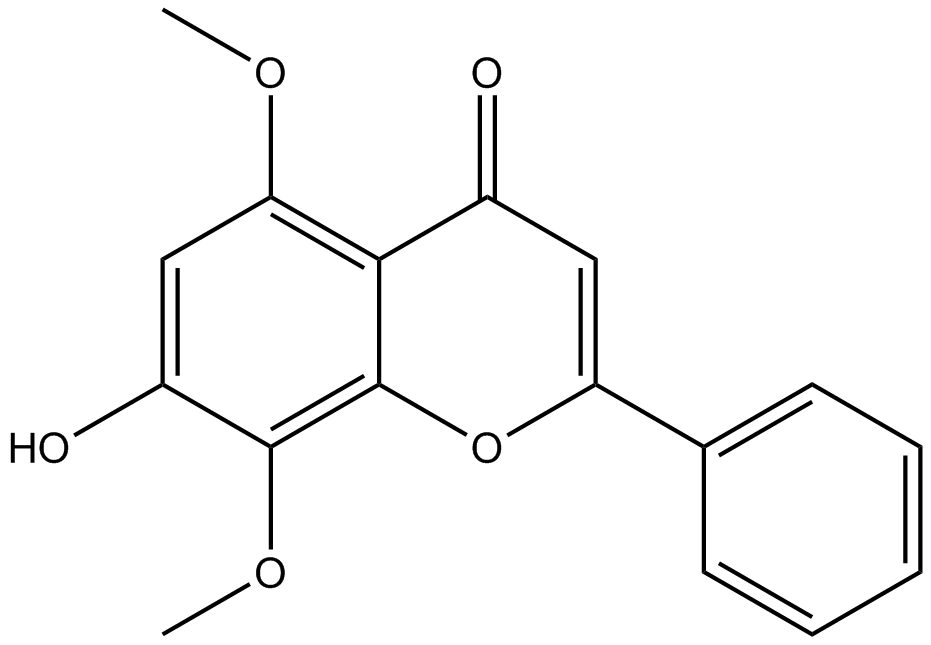 |
| MOL012245 | 5322074 | 5,7,4'-trihydroxy-6-methoxyflavanone^a^ | 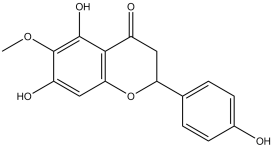 |
| MOL005190 | 440735 | eriodictyol^a^ | 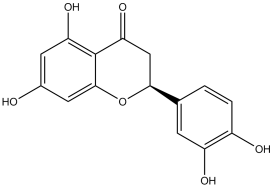 |
| MOL001735 | 5281628 | Dinatin^a^ | 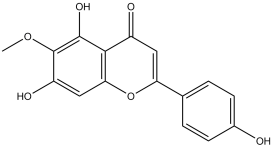 |
| MOL002910 | 188308 | Carthamidin^a^ | 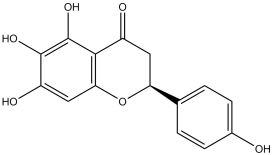 |
| MOL000006 | 5280445 | Luteolin^a,b^ | 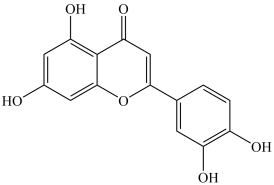 |
| MOL000358 | 222284 | beta-sitosterol (Sitosterol)^a,c,d,e^ | 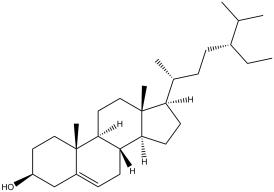 |
| MOL000098 | 5280343 | Quercetin^a,b,d,e^ | 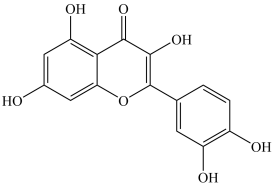 |
| MOL000953 | 131698629 | CLR^a,c,e^ | 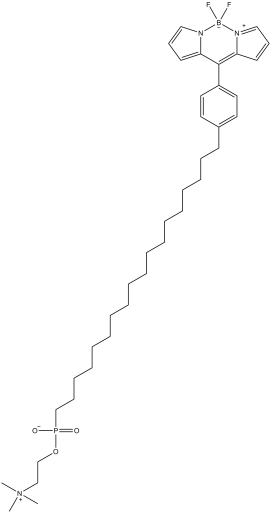 |
| MOL001790 | 5317025 | Linarin^b^ | 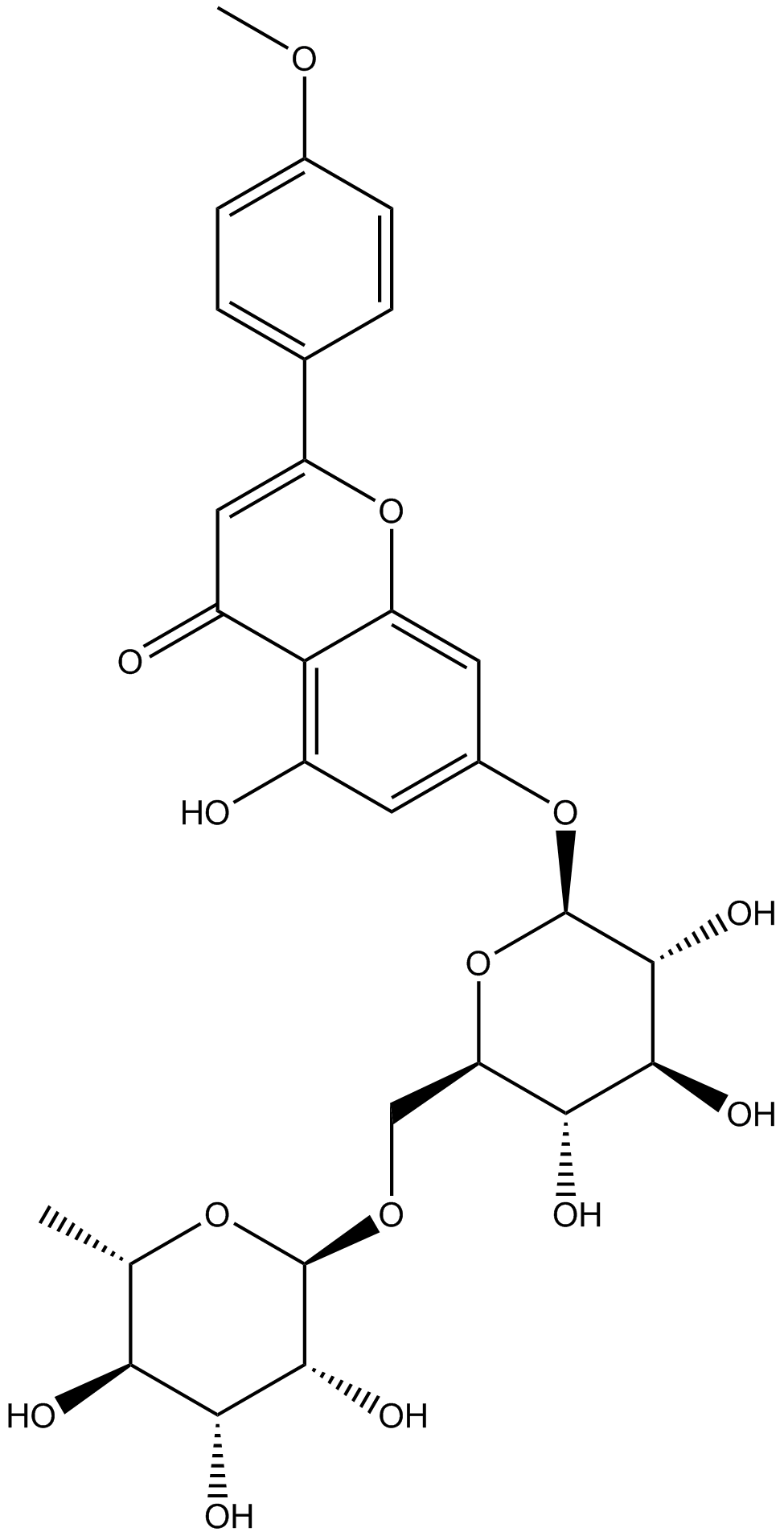 |
| MOL011678 | 6916037 | (3S,8S,9S,10R,13R,14S,17R)-17-[(1S,4R)-4-ethyl-1,5-dimethylhexyl]-10,13-dimethyl-2,3,4,7,8,9,11,12,14,15,16,17-dodecahydro-1H-cyclopenta[a]phenanthren-3-ol^b^ | 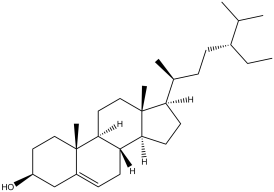 |
| MOL003044 | 5280666 | Chryseriol^b^ | 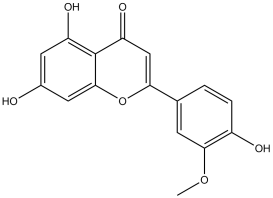 |
| MOL012216 | 12311086 | norlobelanine^b^ | 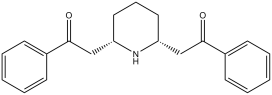 |
| MOL002881 | 5281612 | Diosmetin^b^ | 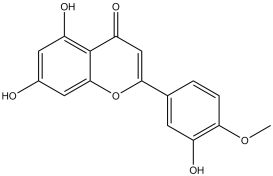 |
| MOL009009 | 181681 | (+)-medioresinol (40957-99-1)^b,e^ | 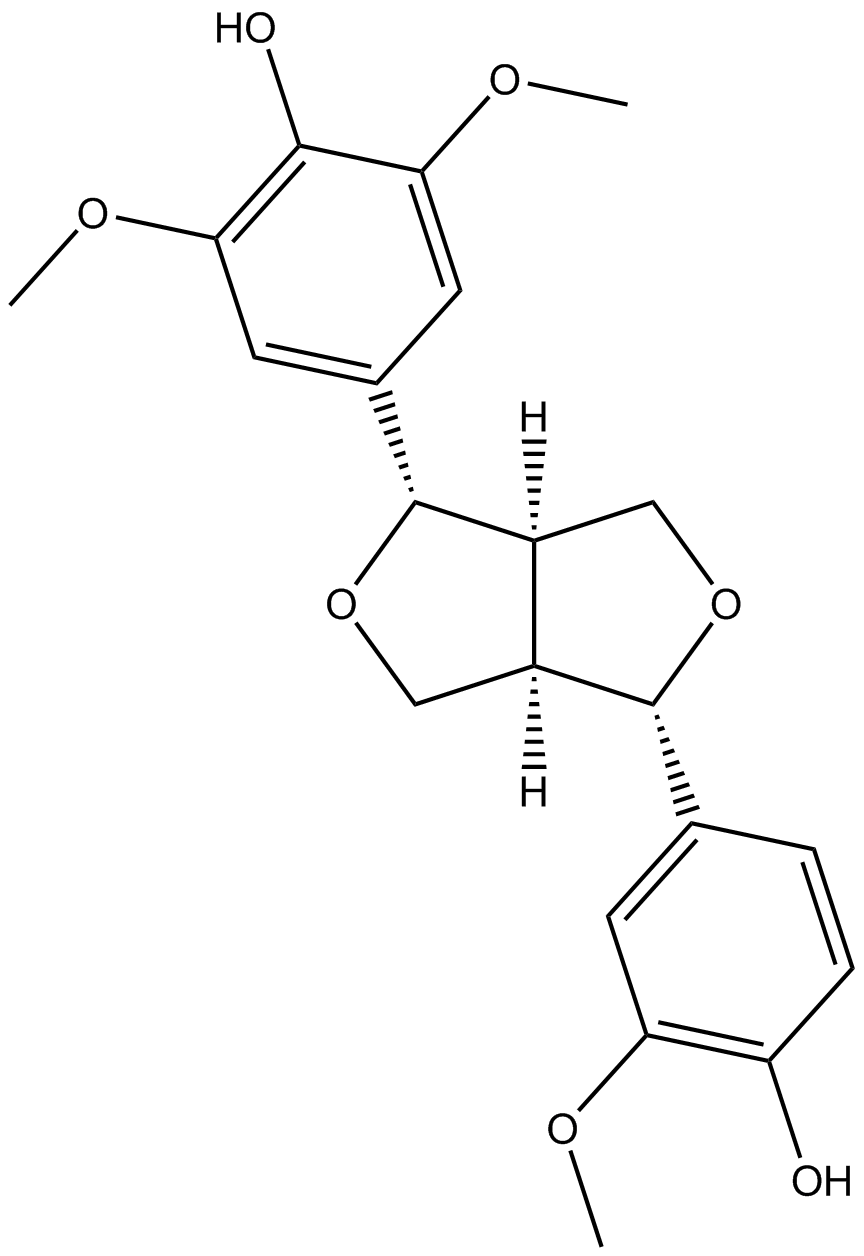 |
| MOL012209 | 688025 | 2-[(2R,6S)-6-[(2R)-2-hydroxy-2-phenylethyl]-1-methylpiperidin-2-yl]-1-phenylethanone^b^ | 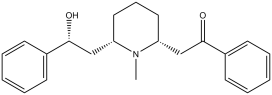 |
| MOL000422 | 5280863 | kaempferol^b^ | 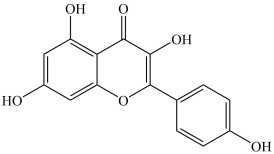 |
| MOL001689 | 5280442 | acacetin^b^ | 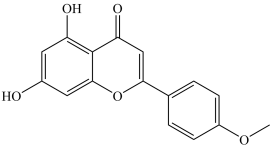 |
| MOL002341 | 72281 | Hesperetin^b^ | 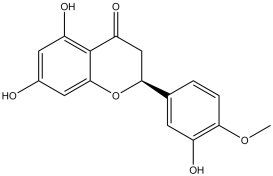 |
| MOL012208 | 442647 | Lobelanine^b^ | 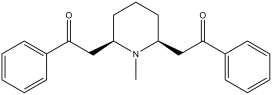 |
| MOL005530 | 5318214 | Hydroxygenkwanin^b^ | 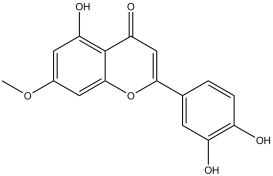 |
| MOL012225 | 11281091 | 2alpha-(3-Methoxy-4-hydroxyphenyl)-5-(3-hydroxypropyl)-7-methoxy-2,3-dihydrobenzofuran-3beta-methanol 5-acetate^b^ | 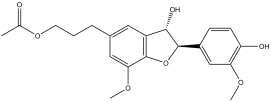 |
| MOL012207 | 442646 | lobelanidine^b^ | 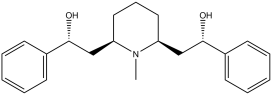 |
| MOL008121 | 5319879 | 2-Monoolein^c^ | 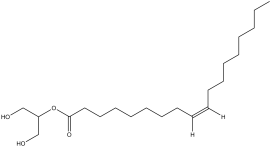 |
| MOL001494 | 5282184 | Mandenol^c^ | 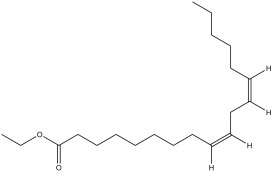 |
| MOL001323 | 9548595 | Sitosterol alpha1^c^ | 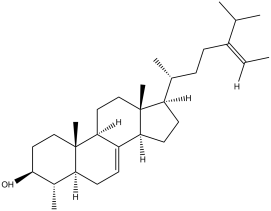 |
| MOL008118 | 46173943 | Coixenolide | 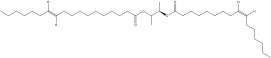 |
| MOL001670 | 10514946 | 2-methoxy-3-methyl-9,10-anthraquinone^d^ | 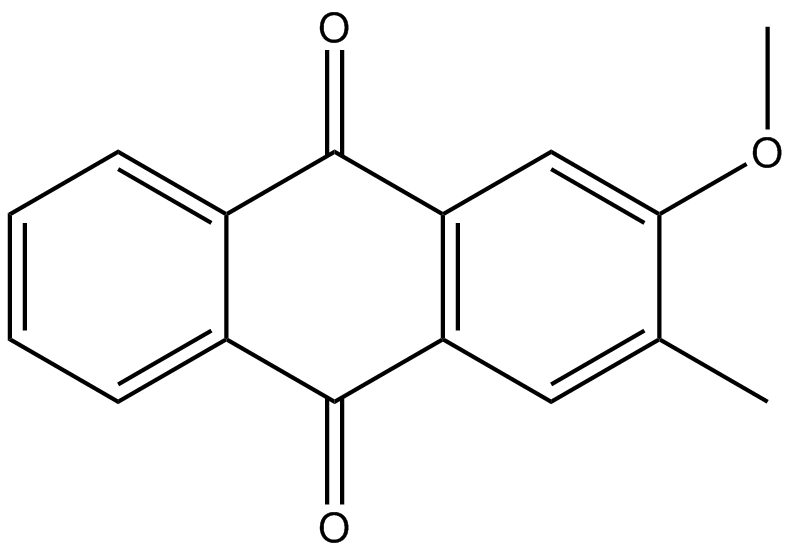 |
| MOL001659 | 5281330 | Poriferasterol^d^ | 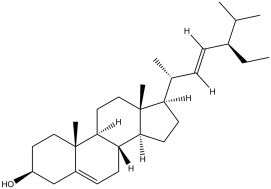 |
| MOL002773 | 5280489 | beta-carotene^e^ | 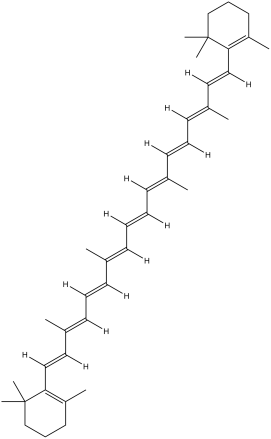 |
| MOL007356 | 73419 | solanocapsine^e^ | 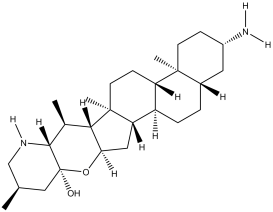 |
| MOL000546 | 99474 | diosgenin^e^ | 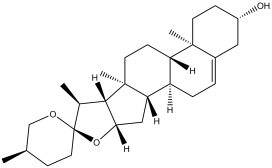 |
| MOL006859 | 31307 | Volon^f^ | 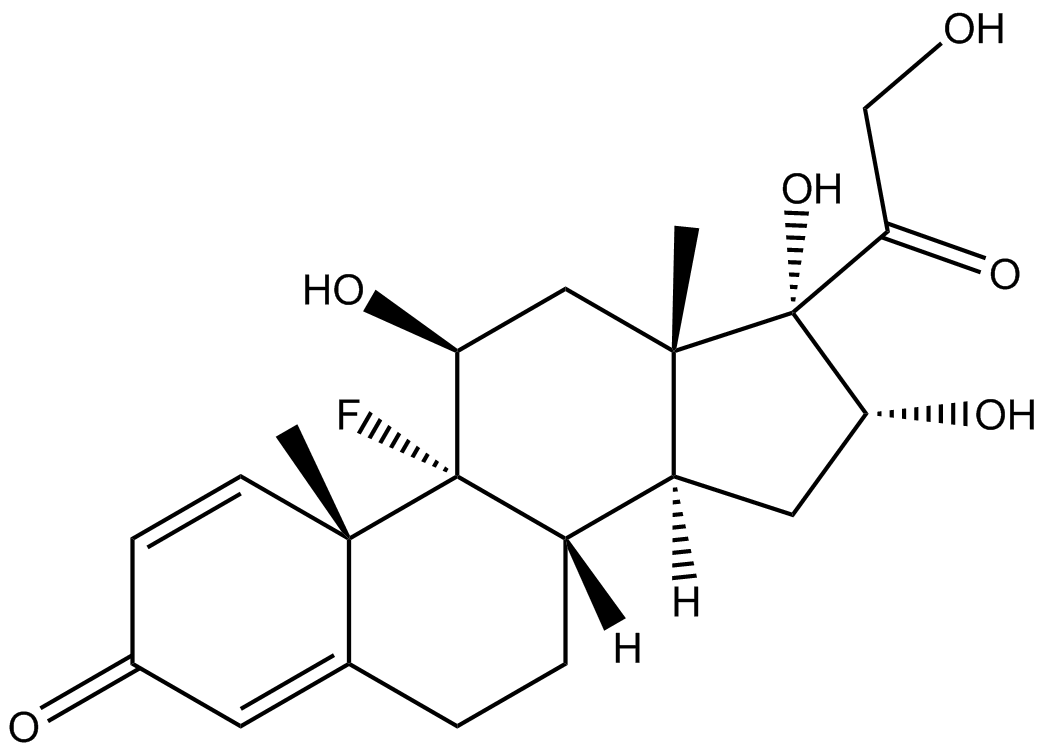 |
| BATMAN001 | 11382599 | Lochnericine^b^ | 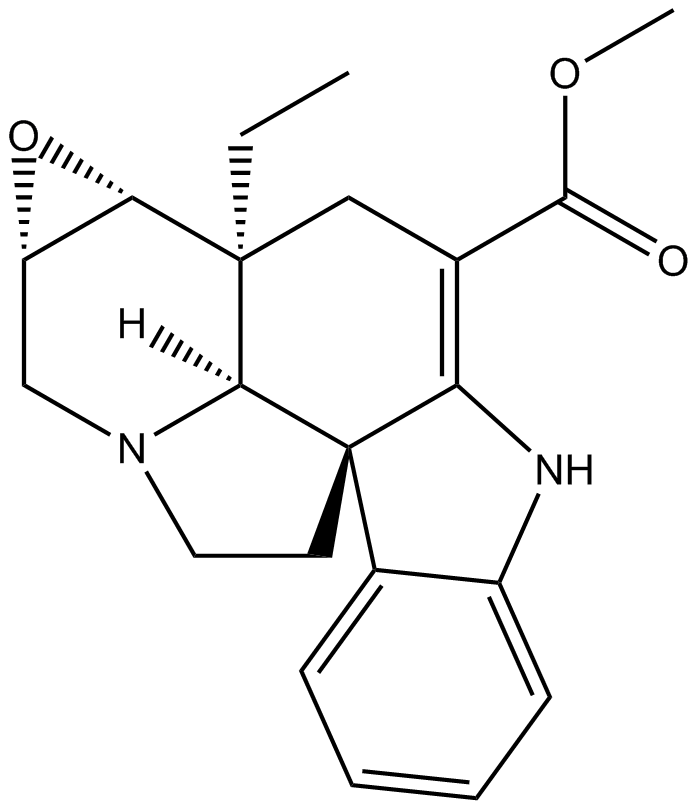 |
| BATMAN002 | 15953621 | Radicamine B^b^ | 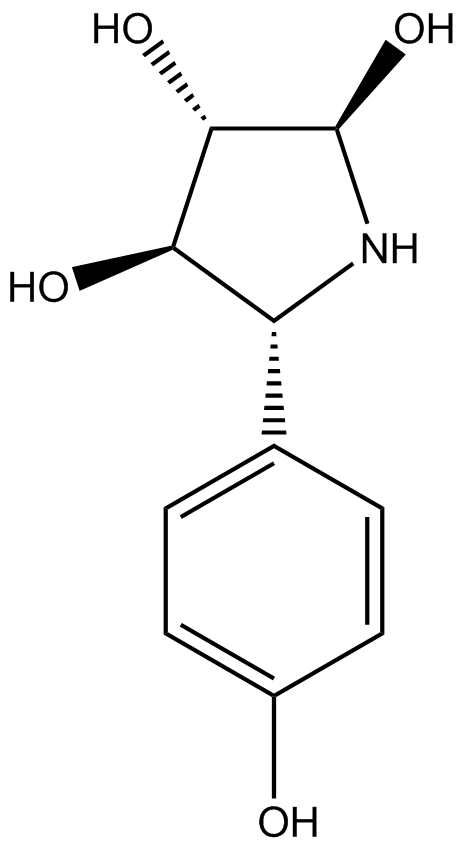 |

CID, Compound CID from PubChem database; The molecular name comes from TCMSP and BATMAN database. a, Scutellariae Barbatae Herba; b, Lobeliae Chinensis Herba; c, Coicis Semen; d, Hedyotis Diffusae Herba; e, Solanum Nigrum Linn; f, Herba Solani Lyrati. The 2D structure of each molecule was drawn by ChemBioDraw Ultra 14.0 software.
