# Supplementary figures and images for: Qingfei Jiedu decoction inhibits PD-L1 expression in lung adenocarcinoma based on network pharmacology analysis, molecular docking and experimental verification
Source: Front Pharmacol. 2022 Aug 22;13:897966. doi: 10.3389/fphar.2022.897966 (PMC9454399; doi:10.3389/fphar.2022.897966)

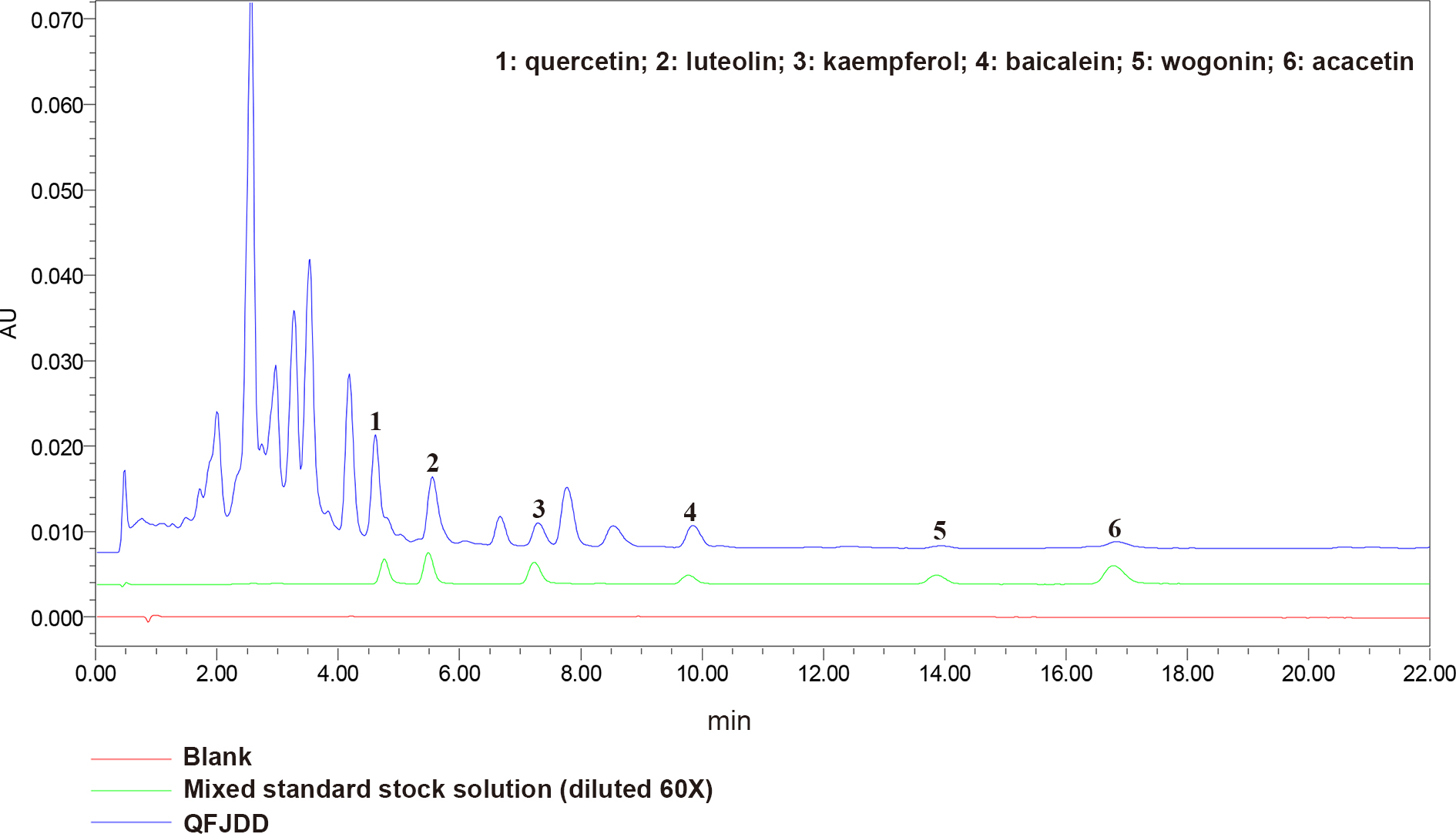

Supplement: Supplementary file 1 [file DataSheet1.ZIP › Supplementary Table and Figure/Supplementary Figure S1.jpg]
